# Supplementary material for: Polyketide synthase gene clusters in the ericoid mycorrhizal fungus Oidiodendron maius: genome-wide prediction, expression profiling, and functional characterization of knockout mutants
Source: Front Plant Sci. 2026 Sep 16;17:1924430. doi: 10.3389/fpls.2026.1924430 (PMC13623903; doi:10.3389/fpls.2026.1924430)
Supplement: Supplementary file 1 [file SupplementaryFile1.zip › Supplementary Materials/Supplementary Folder 1.zip!/Oidma1_AssemblyScaffolds_Repeatmasked/knownclusterblast/cluster42/jgi.p_Oidma1_171109_mibig_hits.html]

| MIBiG Protein | Description | MIBiG Cluster | MiBiG Product | % ID | % Coverage | BLAST Score | E-value |
| --- | --- | --- | --- | --- | --- | --- | --- |
| BAE56606.1 |  | BGC0001123 | Aspirochlorine\_biosynthetic\_gene\_cluster | 40 | 102.358783364 | 1103 | 0.0 |
| EAU36744.1 | predicted\_protein | BGC0000292 | Acetylaranotin\_biosynthetic\_gene\_cluster | 45 | 62.3836126629 | 809 | 7.3e-234 |
| EAL92291.2 | nonribosomal\_peptide\_synthase\_GliP-like,\_putative | BGC0000372 | Hexadehydro-astechrome\_(HAS)\_biosynthetic\_gene\_cluster | 29 | 100.86902545 | 560 | 5.9e-159 |
| CCE28989.1 | non-ribosomal\_peptide\_synthetase | BGC0001365 | Clapurines\_biosynthetic\_gene\_cluster | 28 | 86.2197392924 | 453 | 1.3e-126 |
| AFJ14794.1 | PlpE | BGC0000403 | Pelgipeptin\_biosynthetic\_gene\_cluster | 26 | 78.6468032278 | 383 | 9.9e-106 |
| AAS92545.1 | SirP | BGC0001044 | Sirodesmin\_biosynthetic\_gene\_cluster | 33 | 45.3134698945 | 380 | 8.4e-105 |
| ACZ55944.1 | non-ribosomal\_peptide\_synthetase | BGC0000302 | Anabaenopeptin\_biosynthetic\_gene\_cluster | 26 | 86.2197392924 | 362 | 2.4e-99 |
| ACA09733.2 | fusaricidin\_synthetase | BGC0000358 | Fusaricidin\_biosynthetic\_gene\_cluster | 26 | 87.7094972067 | 361 | 4e-99 |
| BAH43870.1 | putative\_linear\_pentadecapeptide\_gramicidin\_synthetase\_LgrB | BGC0000367 | Gramicidin\_biosynthetic\_gene\_cluster | 25 | 94.5996275605 | 353 | 1.1e-96 |
| CDG17985.1 | Putative\_Ornithine\_racemase\_(fragment) | BGC0000464 | Xenoamicins\_biosynthetic\_gene\_cluster | 25 | 91.1855990068 | 347 | 6e-95 |
| AEA30273.1 | peptide\_synthetase | BGC0000429 | Skyllamycin\_biosynthetic\_gene\_cluster | 26 | 88.0198634389 | 345 | 3.9e-94 |
| KPN93065.1 | NunD | BGC0001416 | Nunapeptin\_/\_nunamycin\_biosynthetic\_gene\_cluster | 27 | 81.9366852886 | 344 | 6.6e-94 |
| AHD05678.1 | nonribosomal\_peptide\_ligase\_subunit | BGC0000402 | Paenilarvins\_biosynthetic\_gene\_cluster | 25 | 88.1440099317 | 344 | 8.7e-94 |
| AHF21228.1 | TriD | BGC0000449 | Tridecaptin\_biosynthetic\_gene\_cluster | 25 | 88.0198634389 | 343 | 1.5e-93 |
| CAB38517.1 | CDA\_peptide\_synthetase\_II | BGC0000315 | Calcium-dependent\_antibiotic\_biosynthetic\_gene\_cluster | 26 | 88.2681564246 | 342 | 3.3e-93 |
| AAC06348.1 | bacitracin\_synthetase\_3 | BGC0000310 | Bacitracin\_biosynthetic\_gene\_cluster | 25 | 86.8404717567 | 341 | 7.4e-93 |
| ABV79987.1 | ApnC | BGC0000301 | Anabaenopeptin\_biosynthetic\_gene\_cluster | 26 | 86.9646182495 | 341 | 7.4e-93 |
| AAU39360.1 | lichenysin\_synthase\_LchAB | BGC0000381 | Lichenysin\_biosynthetic\_gene\_cluster | 26 | 88.5785226567 | 338 | 3.6e-92 |
| CBZ42146.1 | putative\_non-ribosomal\_peptide\_synthetase | BGC0001117 | Himastatin\_biosynthetic\_gene\_cluster | 26 | 87.0887647424 | 337 | 6.2e-92 |
| AJF34464.1 | Txo2 | BGC0001207 | Teixobactin\_biosynthetic\_gene\_cluster | 26 | 88.5164494103 | 337 | 1.1e-91 |
| AAO23334.1 | NcpB | BGC0000397 | Nostocyclopeptide\_biosynthetic\_gene\_cluster | 23 | 98.696461825 | 336 | 1.8e-91 |
| CAC48360.1 | peptide\_synthetase | BGC0000311 | Balhimycin\_biosynthetic\_gene\_cluster | 28 | 67.7839851024 | 333 | 1.2e-90 |
| AOA33123.1 | nonribosomal\_peptide\_synthetase | BGC0001346 | Bananamides\_biosynthetic\_gene\_cluster | 27 | 81.9987585351 | 333 | 1.5e-90 |
| CAE02631.1 | surfactin\_synthetase\_B | BGC0000433 | Surfactin\_biosynthetic\_gene\_cluster | 25 | 83.7988826816 | 332 | 2.6e-90 |
| AAY37653.1 | Amino\_acid\_adenylation | BGC0000437 | Syringomycin\_biosynthetic\_gene\_cluster | 27 | 80.1365611421 | 330 | 9.9e-90 |
| CBJ90082.1 | Non\_Ribosomal\_peptide\_synthetase\_(-succinylbenzoate--CoA\_ligase) | BGC0001132 | Xenotetrapeptide\_biosynthetic\_gene\_cluster | 27 | 87.026691496 | 329 | 2.9e-89 |
| AGM16412.1 | paenibacterin\_synthetase\_A | BGC0000400 | Paenibacterin\_biosynthetic\_gene\_cluster | 25 | 87.3370577281 | 329 | 2.9e-89 |
| AEP18656.1 | WAPS1 | BGC0000461 | WAP-8294A2\_(lotilibcin)\_biosynthetic\_gene\_cluster | 25 | 90.5648665425 | 329 | 2.9e-89 |
| CAB38518.1 | CDA\_peptide\_synthetase\_I | BGC0000315 | Calcium-dependent\_antibiotic\_biosynthetic\_gene\_cluster | 26 | 87.9577901924 | 329 | 2.9e-89 |
| AJF34463.1 | Txo1 | BGC0001207 | Teixobactin\_biosynthetic\_gene\_cluster | 26 | 83.4885164494 | 326 | 1.4e-88 |
| BAH43871.1 | truncated\_linear\_pentadecapeptide\_gramicidin\_synthetase\_LgrC | BGC0000367 | Gramicidin\_biosynthetic\_gene\_cluster | 27 | 63.8733705773 | 325 | 4.2e-88 |
| AEH41794.1 | HrmP | BGC0000374 | Hormaomycin\_biosynthetic\_gene\_cluster | 25 | 87.5853507138 | 325 | 4.2e-88 |
| CAE15637.1 |  | BGC0001128 | Luminmide\_biosynthetic\_gene\_cluster | 27 | 81.1918063315 | 324 | 5.5e-88 |
| ACA97576.1 | PmxA | BGC0000408 | Polymyxin\_biosynthetic\_gene\_cluster | 24 | 81.6883923029 | 324 | 7.1e-88 |
| ABF87031.1 | non-ribosomal\_peptide\_synthetase/polyketide\_synthase | BGC0000393 | Myxoprincomide\_biosynthetic\_gene\_cluster | 26 | 87.8336436996 | 322 | 2.7e-87 |
| ABS74207.1 | FenC | BGC0001095 | Fengycin\_biosynthetic\_gene\_cluster | 26 | 85.6610800745 | 322 | 2.7e-87 |
| ctg1\_orf10 |  | BGC0000321 | Chaetocin\_biosynthetic\_gene\_cluster | 28 | 60.7076350093 | 322 | 3.5e-87 |
| AHZ34233.1 | CifB | BGC0000323 | Cichofactin\_biosynthetic\_gene\_cluster | 26 | 79.6399751707 | 321 | 6e-87 |
| ANQ43349.1 | non-ribosomal\_peptide\_synthetase | BGC0001347\_c2 | Poaeamide\_B\_biosynthetic\_gene\_cluster | 26 | 87.5853507138 | 321 | 6e-87 |
| ALV82356.1 | CDA\_peptide\_synthetase\_I | BGC0001370 | Lipopeptide\_8D1-1\_/\_lipopeptide\_8D1-2\_biosynthetic\_gene\_cluster | 26 | 87.8336436996 | 320 | 1e-86 |
| AGE25480.1 | amino\_acid\_adenylation\_protein | BGC0001208\_c2 | Poaeamide\_biosynthetic\_gene\_cluster | 26 | 88.0819366853 | 319 | 2.3e-86 |
| ABW00331.1 | amino\_acid\_adenylation\_domain | BGC0000333 | Cyclomarin\_biosynthetic\_gene\_cluster | 26 | 82.991930478 | 318 | 5.1e-86 |
| AAZ55900.1 | non-ribosomal\_peptide\_synthase:Amino\_acid\_adenylation | BGC0000359 | Fuscachelin\_biosynthetic\_gene\_cluster | 26 | 87.5232774674 | 318 | 5.1e-86 |
| AEI58865.1 | peptide\_synthetase | BGC0000455 | Vancomycin\_biosynthetic\_gene\_cluster | 25 | 92.1787709497 | 316 | 1.9e-85 |
| AAY91421.3 | non-ribosomal\_peptide\_synthetase\_OfaC | BGC0000399 | Orfamide\_biosynthetic\_gene\_cluster | 28 | 64.9286157666 | 316 | 2.5e-85 |
| AFH75330.1 | nonribosomal\_peptide\_synthetase | BGC0000398 | Orfamide\_biosynthetic\_gene\_cluster | 27 | 67.5356921167 | 315 | 4.3e-85 |
| CDG17980.1 | Putative\_Ornithine\_racemase\_(fragment) | BGC0000464 | Xenoamicins\_biosynthetic\_gene\_cluster | 29 | 67.1011793917 | 314 | 5.6e-85 |
| ABS74206.1 | FenD | BGC0001095 | Fengycin\_biosynthetic\_gene\_cluster | 26 | 80.9435133457 | 314 | 7.4e-85 |
| AAU34202.1 | mannopeptimycin\_peptide\_synthetase\_MppA | BGC0000388 | Mannopeptimycin\_biosynthetic\_gene\_cluster | 26 | 74.7361887027 | 313 | 1.3e-84 |
| CAA11794.1 | PCZA363.3 | BGC0000322 | Chloroeremomycin\_biosynthetic\_gene\_cluster | 28 | 63.9354438237 | 312 | 2.8e-84 |
| AEZ51516.1 | PmxA | BGC0001153 | Polymyxin\_biosynthetic\_gene\_cluster | 24 | 81.7504655493 | 311 | 4.8e-84 |
| BAC67536.1 | arthrofactin\_synthetase\_C | BGC0000305 | Arthrofactin\_biosynthetic\_gene\_cluster | 26 | 79.8882681564 | 311 | 4.8e-84 |
| AAO56329.1 | non-ribosomal\_peptide\_synthetase\_SyfB | BGC0000435 | Syringafactin\_biosynthetic\_gene\_cluster | 27 | 80.69522036 | 311 | 6.2e-84 |
| CDG17982.1 | Non-ribosomal\_peptide\_synthetase | BGC0000464 | Xenoamicins\_biosynthetic\_gene\_cluster | 25 | 88.330229671 | 311 | 8.1e-84 |
| CAG29032.1 | nonribosomal\_peptide\_synthetase\_(modules\_3\_to\_6) | BGC0001023 | Myxochromide\_biosynthetic\_gene\_cluster | 25 | 87.2749844817 | 310 | 1.1e-83 |
| ACA97580.1 | PmxE | BGC0000408 | Polymyxin\_biosynthetic\_gene\_cluster | 24 | 88.9509621353 | 310 | 1.4e-83 |
| ALV82384.1 | CDA\_peptide\_synthetase\_II | BGC0001370 | Lipopeptide\_8D1-1\_/\_lipopeptide\_8D1-2\_biosynthetic\_gene\_cluster | 25 | 88.6405959032 | 309 | 1.8e-83 |
| AGE11899.1 | nonribosomal\_peptide\_synthetase | BGC0000366 | Gobichelin\_biosynthetic\_gene\_cluster | 27 | 88.5785226567 | 309 | 2.4e-83 |
| ABQ96384.2 | fusaricidin\_synthetase | BGC0001152 | Fusaricidin\_biosynthetic\_gene\_cluster | 25 | 87.3991309745 | 308 | 4e-83 |
| sln7 | nonribosomal\_peptide\_synthetase | BGC0001230 | Salinamides\_biosynthetic\_gene\_cluster | 25 | 89.6337678461 | 308 | 5.3e-83 |
| AEW31020.1 | plipastatin\_synthetase | BGC0000407 | Plipastatin\_biosynthetic\_gene\_cluster | 25 | 88.330229671 | 308 | 5.3e-83 |
| AEG64697.1 | LpmC | BGC0000379 | Laspartomycin\_biosynthetic\_gene\_cluster | 25 | 88.6405959032 | 308 | 5.3e-83 |
| AHJ31215.1 | Long-chain-fatty-acid--CoA\_ligase | BGC0000430 | Spumigin\_biosynthetic\_gene\_cluster | 24 | 89.0130353818 | 307 | 9e-83 |
| AEG64698.1 | LpmD | BGC0000379 | Laspartomycin\_biosynthetic\_gene\_cluster | 26 | 80.3227808814 | 306 | 2e-82 |
| AGM16413.1 | paenibacterin\_synthetase\_B | BGC0000400 | Paenibacterin\_biosynthetic\_gene\_cluster | 24 | 88.0819366853 | 305 | 2.6e-82 |
| AEZ51520.1 | PmxE | BGC0001153 | Polymyxin\_biosynthetic\_gene\_cluster | 24 | 87.026691496 | 305 | 2.6e-82 |
| EFE73313.1 | nonribosomal\_peptide\_synthetase | BGC0000431 | Stenothricin\_biosynthetic\_gene\_cluster | 26 | 76.1638733706 | 305 | 3.4e-82 |
| AEH41793.1 | HrmO | BGC0000374 | Hormaomycin\_biosynthetic\_gene\_cluster | 27 | 63.8112973309 | 305 | 3.4e-82 |
| AEU11006.1 | NpnB | BGC0001029 | Nostophycin\_biosynthetic\_gene\_cluster | 25 | 66.4183736809 | 304 | 5.8e-82 |
| AFP87549.1 | NrpS | BGC0001135 | Bicornutin\_biosynthetic\_gene\_cluster | 26 | 79.6399751707 | 304 | 7.6e-82 |
| ACZ55942.1 | non-ribosomal\_peptide\_synthetase | BGC0000302 | Anabaenopeptin\_biosynthetic\_gene\_cluster | 27 | 64.8044692737 | 304 | 1e-81 |
| AJV88377.1 | MfnE | BGC0001214 | Marformycins\_biosynthetic\_gene\_cluster | 26 | 79.6399751707 | 303 | 1.3e-81 |
| AHZ34243.1 | CipF | BGC0001389 | Cichopeptin\_biosynthetic\_gene\_cluster | 26 | 80.7572936065 | 303 | 1.3e-81 |
| AJM89738.1 | PmxE | BGC0001192 | Colistin\_biosynthetic\_gene\_cluster | 24 | 87.3370577281 | 303 | 1.7e-81 |
| CBW45647.1 | PI\_synthetase\_3\_and\_4 | BGC0000952 | Pristinamycin\_biosynthetic\_gene\_cluster | 26 | 82.3091247672 | 302 | 2.2e-81 |
| AKC91849.1 | nonribosomal\_peptide\_synthetase | BGC0001414 | Griselimycin\_biosynthetic\_gene\_cluster | 25 | 90.0062073246 | 302 | 2.2e-81 |
| CAD17793.1 | probable\_non\_ribosomal\_peptide\_synthetase\_protein | BGC0001363 | Ralsolamycin\_biosynthetic\_gene\_cluster | 27 | 88.2681564246 | 301 | 4.9e-81 |
| CAM56770.1 |  | BGC0000354 | Friulimicin\_biosynthetic\_gene\_cluster | 27 | 73.9913097455 | 300 | 8.4e-81 |
| ALV86867.1 | Tlo21 | BGC0001406 | Telomycin\_biosynthetic\_gene\_cluster | 26 | 87.5232774674 | 299 | 1.9e-80 |
| ADJ63842.1 | non-ribosomal\_peptide\_synthetase\_protein | BGC0000424 | Serobactins\_biosynthetic\_gene\_cluster | 26 | 80.0744878957 | 299 | 1.9e-80 |
| AEH59100.1 | amino\_acid\_adenylation\_domain-containing\_protein/NRPS | BGC0000385 | Lysobactin\_biosynthetic\_gene\_cluster | 28 | 66.1080074488 | 299 | 2.5e-80 |
| CAJ77696.1 | MPS2\_protein | BGC0000363 | Glycopeptidolipid\_biosynthetic\_gene\_cluster | 29 | 58.2247051521 | 299 | 2.5e-80 |
| AAX31558.1 | peptide\_synthetase\_2 | BGC0000336 | Daptomycin\_biosynthetic\_gene\_cluster | 27 | 75.7293606456 | 299 | 2.5e-80 |
| AAO62587.1 | peptide\_sythetase | BGC0001016 | Microcystin\_biosynthetic\_gene\_cluster | 24 | 87.3991309745 | 299 | 3.2e-80 |
| ALG65318.1 | Cal18 | BGC0001297 | WS9326\_biosynthetic\_gene\_cluster | 26 | 87.461204221 | 297 | 7.1e-80 |
| CAJ46692.1 | non\_ribosomal\_peptide\_synthase | BGC0000969 | Chondramide\_biosynthetic\_gene\_cluster | 27 | 59.7144630664 | 296 | 2.1e-79 |
| BAH04162.1 | trsJ | BGC0000450 | Triostin\_A\_biosynthetic\_gene\_cluster | 27 | 66.6666666667 | 295 | 2.7e-79 |
| ABD65958.1 | nonribosomal\_peptide\_synthetase | BGC0000341 | Enduracidin\_biosynthetic\_gene\_cluster | 27 | 75.0465549348 | 295 | 3.5e-79 |
| AJM89735.1 | PmxA | BGC0001192 | Colistin\_biosynthetic\_gene\_cluster | 24 | 82.0608317815 | 294 | 6e-79 |
| CAD91220.1 | putative\_non-ribosomal\_peptide\_synthetase,\_modules\_1-2 | BGC0000289 | A40926\_biosynthetic\_gene\_cluster | 27 | 63.1284916201 | 293 | 1.3e-78 |
| KPN90376.1 | NupC | BGC0001416 | Nunapeptin\_/\_nunamycin\_biosynthetic\_gene\_cluster | 26 | 79.391682185 | 292 | 2.3e-78 |
| AEW31019.1 | plipastatin\_synthetase | BGC0000407 | Plipastatin\_biosynthetic\_gene\_cluster | 27 | 69.8944754811 | 292 | 3e-78 |
| ACM79812.1 | ZmaQ | BGC0001059 | Zwittermycin\_A\_biosynthetic\_gene\_cluster | 26 | 78.2743637492 | 292 | 3e-78 |
| AHZ20774.1 | non-ribosomal\_peptide\_synthase | BGC0000369 | Hassallidins\_biosynthetic\_gene\_cluster | 27 | 64.121663563 | 292 | 3.9e-78 |
| AAY37647.1 | Amino\_acid\_adenylation | BGC0000437 | Syringomycin\_biosynthetic\_gene\_cluster | 25 | 88.3923029174 | 291 | 6.7e-78 |
| AEW31021.1 | plipastatin\_synthetase | BGC0000407 | Plipastatin\_biosynthetic\_gene\_cluster | 27 | 69.4599627561 | 291 | 6.7e-78 |
| AGS77307.1 | NRPS\_modules\_1-2 | BGC0001178 | UK-68,597\_biosynthetic\_gene\_cluster | 25 | 95.7169459963 | 290 | 1.5e-77 |
| CAJ77716.1 | Mps2\_protein | BGC0000364 | Glycopeptidolipid\_biosynthetic\_gene\_cluster | 28 | 58.1626319056 | 289 | 1.9e-77 |
| AED90003.1 | non-ribosomal\_peptide\_synthetase\_ThaB | BGC0000443 | Thanamycin\_biosynthetic\_gene\_cluster | 29 | 66.7287399131 | 289 | 2.5e-77 |
| CAE02630.1 | surfactin\_synthetase\_A | BGC0000433 | Surfactin\_biosynthetic\_gene\_cluster | 25 | 80.8814400993 | 288 | 4.3e-77 |
| AAU39359.1 | lichenysin\_synthase\_LchAA | BGC0000381 | Lichenysin\_biosynthetic\_gene\_cluster | 25 | 79.9503414029 | 288 | 5.7e-77 |
| CDG17981.1 | Non-ribosomal\_peptide\_synthetase | BGC0000464 | Xenoamicins\_biosynthetic\_gene\_cluster | 30 | 52.5139664804 | 288 | 5.7e-77 |
| AOA33122.1 | nonribosomal\_peptide\_synthetase | BGC0001346 | Bananamides\_biosynthetic\_gene\_cluster | 29 | 51.7690875233 | 287 | 1.3e-76 |
| AAF15891.2 | NosA | BGC0001028 | Nostopeptolide\_biosynthetic\_gene\_cluster | 26 | 71.8808193669 | 286 | 1.6e-76 |
| AGM14934.1 | xantholysin\_synthetase\_C | BGC0000463 | Xantholysin\_biosynthetic\_gene\_cluster | 27 | 69.2116697703 | 285 | 3.7e-76 |
| AAY37655.1 | Amino\_acid\_adenylation | BGC0000437 | Syringomycin\_biosynthetic\_gene\_cluster | 30 | 51.582867784 | 285 | 3.7e-76 |
| AAO72425.1 | syringopeptin\_synthetase\_C | BGC0000438 | Syringopeptin\_biosynthetic\_gene\_cluster | 31 | 51.582867784 | 285 | 4.8e-76 |
| AIG79243.1 | Hypothetical\_protein | BGC0000419 | Ristomycin\_A\_biosynthetic\_gene\_cluster | 25 | 82.6194909994 | 284 | 6.3e-76 |
| WP\_020635019.1 | hypothetical\_protein | BGC0001211 | Albachelin\_biosynthetic\_gene\_cluster | 28 | 64.7423960273 | 284 | 6.3e-76 |
| AED90002.1 | non-ribosomal\_peptide\_synthetase\_ThaA | BGC0000443 | Thanamycin\_biosynthetic\_gene\_cluster | 27 | 58.8454376164 | 284 | 1.1e-75 |
| CDG17987.1 | Putative\_Ornithine\_racemase\_(fragment) | BGC0000464 | Xenoamicins\_biosynthetic\_gene\_cluster | 29 | 51.7070142768 | 283 | 1.4e-75 |
| CAB15186.3 | siderophore\_2,3-dihydroxybenzoate-glycine-threonine\_trimeric\_ester\_bacillibactin\_synthetase | BGC0000309 | Bacillibactin\_biosynthetic\_gene\_cluster | 25 | 89.6337678461 | 283 | 1.8e-75 |
| AHD05614.1 | putative\_non-ribosomal\_peptide\_ligase/\_polyketide\_synthase\_hybrid | BGC0001033 | Paenilamicin\_biosynthetic\_gene\_cluster | 25 | 64.8044692737 | 282 | 2.4e-75 |
| AFJ23826.1 | WLIP\_synthetase\_C | BGC0000462\_c2 | WLIP\_biosynthetic\_gene\_cluster | 26 | 73.2464307883 | 282 | 2.4e-75 |
| AIE77057.1 | peptide\_synthetase | BGC0000418 | Ristocetin\_biosynthetic\_gene\_cluster | 25 | 82.9298572315 | 282 | 3.1e-75 |
| AGE11898.1 | nonribosomal\_peptide\_synthetase | BGC0000366 | Gobichelin\_biosynthetic\_gene\_cluster | 27 | 71.5704531347 | 282 | 3.1e-75 |
| AEU11003.1 | NpnC | BGC0001029 | Nostophycin\_biosynthetic\_gene\_cluster | 24 | 68.8392302917 | 282 | 4.1e-75 |
| AAT12283.1 | LtxA | BGC0000384 | Lyngbyatoxin\_biosynthetic\_gene\_cluster | 32 | 41.6511483551 | 282 | 4.1e-75 |
| AAO56328.1 | non-ribosomal\_peptide\_synthetase\_SyfA | BGC0000435 | Syringafactin\_biosynthetic\_gene\_cluster | 29 | 50.0310366232 | 281 | 5.3e-75 |
| AGM16414.1 | paenibacterin\_synthetase\_C | BGC0000400 | Paenibacterin\_biosynthetic\_gene\_cluster | 26 | 70.5152079454 | 281 | 5.3e-75 |
| ctg1\_orf003 |  | BGC0000334 | Cyclosporin\_biosynthetic\_gene\_cluster | 25 | 77.0328988206 | 280 | 9e-75 |
| CAD55498.1 | CDA\_peptide\_synthetase\_III | BGC0000315 | Calcium-dependent\_antibiotic\_biosynthetic\_gene\_cluster | 28 | 61.1421477343 | 280 | 9e-75 |
| AHI59110.1 | locillomycin\_synthase\_C | BGC0001005 | Locillomycin\_biosynthetic\_gene\_cluster | 24 | 77.8398510242 | 280 | 1.5e-74 |
| ALV82388.1 | CDA\_peptide\_synthetase\_III | BGC0001370 | Lipopeptide\_8D1-1\_/\_lipopeptide\_8D1-2\_biosynthetic\_gene\_cluster | 28 | 61.7008069522 | 280 | 1.5e-74 |
| AAC06346.1 | bacitracin\_synthetase\_1 | BGC0000310 | Bacitracin\_biosynthetic\_gene\_cluster | 25 | 80.8193668529 | 280 | 1.5e-74 |
| AIE77058.1 | peptide\_synthetase\_module\_3 | BGC0000418 | Ristocetin\_biosynthetic\_gene\_cluster | 27 | 65.8597144631 | 279 | 2.6e-74 |
| CAK15814.1 | putative\_non-ribosomal\_peptide\_synthetase,\_terminal\_component | BGC0000344 | Entolysin\_biosynthetic\_gene\_cluster | 27 | 65.6114214773 | 279 | 2.6e-74 |
| AAX31559.1 | peptide\_synthetase\_3 | BGC0000336 | Daptomycin\_biosynthetic\_gene\_cluster | 32 | 40.0372439479 | 279 | 3.4e-74 |
| AEA30272.1 | peptide\_synthetase | BGC0000429 | Skyllamycin\_biosynthetic\_gene\_cluster | 26 | 70.2669149597 | 278 | 4.5e-74 |
| AHB82071.1 | non\_ribosomal\_peptide\_synthetase | BGC0001231 | Microsclerodermins\_biosynthetic\_gene\_cluster | 26 | 65.1148355059 | 278 | 5.9e-74 |
| AAC06347.1 | bacitracin\_synthetase\_2 | BGC0000310 | Bacitracin\_biosynthetic\_gene\_cluster | 24 | 88.2681564246 | 278 | 5.9e-74 |
| CAD91211.1 | putative\_non-ribosomal\_peptide\_synthetase,\_module\_7 | BGC0000289 | A40926\_biosynthetic\_gene\_cluster | 25 | 83.3022967101 | 277 | 7.6e-74 |
| AEG64696.1 | LpmB | BGC0000379 | Laspartomycin\_biosynthetic\_gene\_cluster | 28 | 63.3767846058 | 277 | 7.6e-74 |
| AGS77308.1 | NRPS\_module\_3 | BGC0001178 | UK-68,597\_biosynthetic\_gene\_cluster | 27 | 57.7281191806 | 277 | 1.3e-73 |
| AHH53506.1 | non-ribosomal\_peptide\_synthetase | BGC0000439 | Taromycin\_biosynthetic\_gene\_cluster | 28 | 60.7076350093 | 276 | 1.7e-73 |
| AJV88375.1 | MfnC | BGC0001214 | Marformycins\_biosynthetic\_gene\_cluster | 33 | 46.4307883302 | 276 | 1.7e-73 |
| AAM80539.1 | StaA | BGC0000290 | A47934\_biosynthetic\_gene\_cluster | 24 | 94.3513345748 | 276 | 2.2e-73 |
| AAO72424.1 | syringopeptin\_synthetase\_B | BGC0000438 | Syringopeptin\_biosynthetic\_gene\_cluster | 30 | 51.2725015518 | 275 | 2.9e-73 |
| CDG17986.1 | Non-ribosomal\_peptide\_synthetase | BGC0000464 | Xenoamicins\_biosynthetic\_gene\_cluster | 30 | 51.7070142768 | 275 | 2.9e-73 |
| CCJ67640.1 | TaaE | BGC0000447 | Tolaasin\_biosynthetic\_gene\_cluster | 28 | 55.5555555556 | 275 | 2.9e-73 |
| AIG79242.1 | Hypothetical\_protein | BGC0000419 | Ristomycin\_A\_biosynthetic\_gene\_cluster | 27 | 60.086902545 | 275 | 5e-73 |
| AAT01807.1 | non-ribosomal\_peptide\_synthetase | BGC0000365 | Glycopeptidolipid\_biosynthetic\_gene\_cluster | 28 | 57.2936064556 | 275 | 5e-73 |
| ALD82526.1 | non-ribosomal\_peptide\_synthase | BGC0001212 | Nannocystin\_biosynthetic\_gene\_cluster | 28 | 51.2725015518 | 274 | 6.5e-73 |
| AFH75322.1 | nonribosomal\_peptide\_synthetase | BGC0000425 | Sessilin\_biosynthetic\_gene\_cluster | 27 | 55.617628802 | 274 | 6.5e-73 |
| AIW82283.1 | PuwF | BGC0001125 | Puwainaphycins\_biosynthetic\_gene\_cluster | 24 | 81.0055865922 | 274 | 1.1e-72 |
| CAE52339.1 | Polyketide\_non-ribosomal\_peptide\_synthase | BGC0001088 | Albicidin\_biosynthetic\_gene\_cluster | 26 | 76.1018001241 | 273 | 1.9e-72 |
| AAZ23077.1 | peptide\_synthetase | BGC0000291 | A54145\_biosynthetic\_gene\_cluster | 27 | 68.0322780881 | 272 | 2.5e-72 |
| AEP18655.1 | WAPS2 | BGC0000461 | WAP-8294A2\_(lotilibcin)\_biosynthetic\_gene\_cluster | 29 | 59.8386095593 | 272 | 2.5e-72 |
| AET98906.1 | putative\_non-ribosomal\_peptide\_synthetase | BGC0000415 | Quinomycin\_biosynthetic\_gene\_cluster | 27 | 58.2867783985 | 272 | 2.5e-72 |
| AGE25479.1 | putative\_non-ribosomal\_peptide\_synthetase | BGC0001208\_c2 | Poaeamide\_biosynthetic\_gene\_cluster | 30 | 50.2172563625 | 272 | 3.2e-72 |
| KPN93064.1 | NupB | BGC0001416 | Nunapeptin\_/\_nunamycin\_biosynthetic\_gene\_cluster | 31 | 51.0862818125 | 272 | 4.2e-72 |
| CAE53351.1 | non-ribosomal\_peptide\_synthetase | BGC0000440 | Teicoplanin\_biosynthetic\_gene\_cluster | 27 | 58.5350713842 | 272 | 4.2e-72 |
| ABS74181.1 | BmyA | BGC0001090 | Bacillomycin\_biosynthetic\_gene\_cluster | 24 | 79.0813159528 | 271 | 5.5e-72 |
| AAD44234.1 | PstB | BGC0000362 | Glycopeptidolipid\_biosynthetic\_gene\_cluster | 28 | 58.1626319056 | 271 | 5.5e-72 |
| AAY37654.1 | Amino\_acid\_adenylation | BGC0000437 | Syringomycin\_biosynthetic\_gene\_cluster | 29 | 51.2725015518 | 271 | 7.2e-72 |
| AAF08795.1 | MycA | BGC0001103 | Mycosubtilin\_biosynthetic\_gene\_cluster | 24 | 81.5021725636 | 270 | 9.3e-72 |
| CAG15010.1 | peptide\_synthetase,\_module\_3 | BGC0000441 | Teicoplanin\_biosynthetic\_gene\_cluster | 27 | 58.5350713842 | 270 | 9.3e-72 |
| BAB69698.1 | iturin\_A\_synthetase\_A | BGC0001098 | Iturin\_biosynthetic\_gene\_cluster | 24 | 79.0192427064 | 270 | 9.3e-72 |
| AGZ03651.1 | SevB | BGC0000426 | Sevadicin\_biosynthetic\_gene\_cluster | 25 | 59.5903165736 | 270 | 9.3e-72 |
| AHD05615.1 | putative\_non-ribosomal\_peptide\_ligase/\_polyketide\_synthase\_hybrid | BGC0001033 | Paenilamicin\_biosynthetic\_gene\_cluster | 23 | 89.9441340782 | 270 | 1.2e-71 |
| AAZ55899.1 | amino\_acid\_adenylation | BGC0000359 | Fuscachelin\_biosynthetic\_gene\_cluster | 29 | 58.9695841092 | 270 | 1.6e-71 |
| AFH75321.1 | nonribosomal\_peptide\_synthetase | BGC0000425 | Sessilin\_biosynthetic\_gene\_cluster | 25 | 73.1843575419 | 270 | 1.6e-71 |
| AAZ23078.1 | peptide\_synthetase | BGC0000291 | A54145\_biosynthetic\_gene\_cluster | 32 | 37.5543140906 | 269 | 2.1e-71 |
| AHB82058.1 | non\_ribosomal\_peptide\_synthetase | BGC0001019 | Microsclerodermins\_biosynthetic\_gene\_cluster | 27 | 60.0248292986 | 269 | 2.7e-71 |
| AAX31557.1 | peptide\_synthetase\_1 | BGC0000336 | Daptomycin\_biosynthetic\_gene\_cluster | 27 | 64.0595903166 | 267 | 1.3e-70 |
| AFT91378.1 | EcdA | BGC0000338 | Echinocandin\_B\_biosynthetic\_gene\_cluster | 24 | 79.5779019243 | 266 | 1.8e-70 |
| BAH43765.1 | tyrocidine\_synthetase\_II | BGC0000452 | Tyrocidine\_biosynthetic\_gene\_cluster | 25 | 64.6182495345 | 266 | 1.8e-70 |
| CAD29797.1 | peptide\_synthetase | BGC0001015 | Microcystin\_biosynthetic\_gene\_cluster | 23 | 84.4196151459 | 266 | 1.8e-70 |
| BAI63288.1 | putative\_non-ribosomal\_peptide\_synthetase | BGC0000434 | SW-163\_biosynthetic\_gene\_cluster | 27 | 58.2247051521 | 266 | 1.8e-70 |
| AAK81824.1 | peptide\_synthetase | BGC0000326 | Complestatin\_biosynthetic\_gene\_cluster | 28 | 58.8454376164 | 265 | 3e-70 |
| ADG27358.1 | peptide\_synthetase | BGC0000296 | Actinomycin\_biosynthetic\_gene\_cluster | 26 | 84.295468653 | 265 | 3e-70 |
| AHZ34240.1 | CipC | BGC0001389 | Cichopeptin\_biosynthetic\_gene\_cluster | 28 | 50.4655493482 | 265 | 3.9e-70 |
| EWS95124.1 | hypothetical\_protein | BGC0000306 | Arylomycin\_biosynthetic\_gene\_cluster | 28 | 64.8044692737 | 265 | 5.1e-70 |
| AAF99707.2 | syringopeptin\_synthetase | BGC0000438 | Syringopeptin\_biosynthetic\_gene\_cluster | 30 | 52.5139664804 | 264 | 6.7e-70 |
| AHZ34238.1 | CipA | BGC0001389 | Cichopeptin\_biosynthetic\_gene\_cluster | 29 | 51.5207945376 | 264 | 8.7e-70 |
| AGS77310.1 | NRPS\_module\_7 | BGC0001178 | UK-68,597\_biosynthetic\_gene\_cluster | 25 | 82.6815642458 | 264 | 8.7e-70 |
| ACY06285.1 | non-ribosomal\_peptide\_synthetase | BGC0001042 | Sanglifehrin\_A\_biosynthetic\_gene\_cluster | 26 | 60.4593420236 | 264 | 1.1e-69 |
| CAP96445.1 |  | BGC0000420 | Roquefortine\_C\_/\_meleagrin\_biosynthetic\_gene\_cluster | 25 | 74.2396027312 | 264 | 1.1e-69 |
| KPN93063.1 | NupA | BGC0001416 | Nunapeptin\_/\_nunamycin\_biosynthetic\_gene\_cluster | 29 | 50.7759155804 | 264 | 1.1e-69 |
| BAC67535.1 | arthrofactin\_synthetase\_B | BGC0000305 | Arthrofactin\_biosynthetic\_gene\_cluster | 29 | 50.3414028554 | 263 | 1.5e-69 |
| CAY48789.1 | putative\_non-ribosomal\_peptide\_synthetase | BGC0001312 | Viscosin\_biosynthetic\_gene\_cluster | 29 | 57.8522656735 | 263 | 1.5e-69 |
| AFH75320.1 | nonribosomal\_peptide\_synthetase | BGC0000425 | Sessilin\_biosynthetic\_gene\_cluster | 28 | 50.5896958411 | 263 | 1.5e-69 |
| BAF50711.1 | non\_ribosomal\_peptide\_synthetase\_for\_virginiamycin\_S | BGC0001116 | Virginiamycin\_biosynthetic\_gene\_cluster | 25 | 84.4196151459 | 263 | 1.9e-69 |
| AAF08797.1 | MycC | BGC0001103 | Mycosubtilin\_biosynthetic\_gene\_cluster | 25 | 59.4040968343 | 262 | 2.5e-69 |
| AAY93355.1 | non-ribosomal\_peptide\_synthetase\_PvdJ | BGC0000413 | Pyoverdine\_biosynthetic\_gene\_cluster | 24 | 81.8746120422 | 262 | 2.5e-69 |
| AHZ34232.1 | CifA | BGC0000323 | Cichofactin\_biosynthetic\_gene\_cluster | 27 | 62.1353196772 | 262 | 2.5e-69 |
| CDF96614.1 | Long-chain-fatty-acid--CoA\_ligase | BGC0001149 | Brabantamide\_biosynthetic\_gene\_cluster | 24 | 89.1371818746 | 262 | 2.5e-69 |
| AGZ15458.1 | putative\_non-ribosomal\_peptide\_synthetase | BGC0001036 | Polyoxypeptin\_biosynthetic\_gene\_cluster | 27 | 59.3420235878 | 262 | 3.3e-69 |
| ACU71638.1 | amino\_acid\_adenylation\_domain\_protein | BGC0001154 | Cacibiocin\_biosynthetic\_gene\_cluster | 32 | 36.8094351335 | 262 | 4.3e-69 |
| CCJ67639.1 | TaaD | BGC0000447 | Tolaasin\_biosynthetic\_gene\_cluster | 28 | 51.9553072626 | 261 | 5.7e-69 |
| AEF33078.1 | dimodular\_nonribosomal\_peptide\_synthetase | BGC0001039 | Pyridomycin\_biosynthetic\_gene\_cluster | 31 | 37.8646803228 | 260 | 9.7e-69 |
| ABW17376.1 | PsoB | BGC0000411 | Putisolvin\_biosynthetic\_gene\_cluster | 29 | 50.1551831161 | 260 | 9.7e-69 |
| AHZ34242.1 | CipE | BGC0001389 | Cichopeptin\_biosynthetic\_gene\_cluster | 28 | 50.2793296089 | 260 | 1.6e-68 |
| AIE77059.1 | peptide\_synthetase | BGC0000418 | Ristocetin\_biosynthetic\_gene\_cluster | 26 | 63.0043451273 | 259 | 2.2e-68 |
| AIG79241.1 | Hypothetical\_protein | BGC0000419 | Ristomycin\_A\_biosynthetic\_gene\_cluster | 26 | 63.4388578523 | 259 | 2.2e-68 |
| AEC14347.1 | nonribosomal\_peptide\_synthetase | BGC0000377 | Koranimine\_biosynthetic\_gene\_cluster | 26 | 58.1005586592 | 259 | 3.7e-68 |
| ALV86866.1 | Tlo20 | BGC0001406 | Telomycin\_biosynthetic\_gene\_cluster | 25 | 91.930477964 | 259 | 3.7e-68 |
| ABP53497.1 | amino\_acid\_adenylation\_domain | BGC0001041 | Salinosporamide\_biosynthetic\_gene\_cluster | 33 | 37.0577281192 | 258 | 6.3e-68 |
| ABP73646.1 | SalB | BGC0000145 | Salinosporamide\_A\_biosynthetic\_gene\_cluster | 33 | 37.0577281192 | 258 | 6.3e-68 |
| AAK81826.1 | peptide\_synthetase | BGC0000326 | Complestatin\_biosynthetic\_gene\_cluster | 26 | 62.6939788951 | 258 | 6.3e-68 |
| AHF21229.1 | TriE | BGC0000449 | Tridecaptin\_biosynthetic\_gene\_cluster | 25 | 59.0316573557 | 258 | 6.3e-68 |
| AKC91857.1 | nonribosomal\_peptide\_synthetase | BGC0001414 | Griselimycin\_biosynthetic\_gene\_cluster | 26 | 57.7901924271 | 258 | 6.3e-68 |
| WP\_028678149.1 | non-ribosomal\_peptide\_synthetase | BGC0001228 | Retimycin\_biosynthetic\_gene\_cluster | 27 | 57.8522656735 | 258 | 6.3e-68 |
| ABV79988.1 | ApnD | BGC0000301 | Anabaenopeptin\_biosynthetic\_gene\_cluster | 26 | 60.52141527 | 258 | 6.3e-68 |
| ABV79986.1 | ApnB | BGC0000301 | Anabaenopeptin\_biosynthetic\_gene\_cluster | 25 | 67.5356921167 | 257 | 8.2e-68 |
| AAY93354.1 | non-ribosomal\_peptide\_synthetase\_PvdD | BGC0000413 | Pyoverdine\_biosynthetic\_gene\_cluster | 25 | 71.8808193669 | 257 | 8.2e-68 |
| ABH06368.2 | MassB | BGC0000389 | Massetolide\_A\_biosynthetic\_gene\_cluster | 30 | 50.4655493482 | 257 | 8.2e-68 |
| AEH59099.1 | amino\_acid\_adenylation\_domain-containing\_protein/NRPS | BGC0000385 | Lysobactin\_biosynthetic\_gene\_cluster | 28 | 58.5971446307 | 257 | 1.1e-67 |
| AHH53507.1 | non-ribosomal\_peptide\_synthetase | BGC0000439 | Taromycin\_biosynthetic\_gene\_cluster | 24 | 79.8882681564 | 257 | 1.1e-67 |
| EFE73312.1 | nonribosomal\_peptide\_synthetase | BGC0000431 | Stenothricin\_biosynthetic\_gene\_cluster | 26 | 66.7287399131 | 257 | 1.1e-67 |
| CAY48788.1 | putative\_non-ribosomal\_peptide\_synthetase | BGC0001312 | Viscosin\_biosynthetic\_gene\_cluster | 30 | 50.7759155804 | 257 | 1.4e-67 |
| AGS77309.1 | NRPS\_modules\_4-6 | BGC0001178 | UK-68,597\_biosynthetic\_gene\_cluster | 28 | 58.9075108628 | 255 | 4.1e-67 |
| CAD91221.1 | putative\_non-ribosomal\_peptide\_synthetase,\_module\_3 | BGC0000289 | A40926\_biosynthetic\_gene\_cluster | 26 | 58.4109248914 | 255 | 5.3e-67 |
| ACU36654.1 | amino\_acid\_adenylation\_domain\_protein | BGC0000392 | Mirubactin\_biosynthetic\_gene\_cluster | 24 | 78.5847299814 | 255 | 5.3e-67 |
| CAA11795.1 | PCZA363.4 | BGC0000322 | Chloroeremomycin\_biosynthetic\_gene\_cluster | 27 | 61.2662942272 | 255 | 5.3e-67 |
| EDY47118.1 | N-(5-amino-5-carboxypentanoyl)-L-cysteinyl-D-\_valine\_synthase | BGC0000319 | Cephamycin\_C\_biosynthetic\_gene\_cluster | 24 | 71.8808193669 | 253 | 1.2e-66 |
| CAJ14037.1 | peptide\_synthetase\_PhsB | BGC0000406 | Phosphinothricin\_biosynthetic\_gene\_cluster | 33 | 37.9888268156 | 253 | 1.2e-66 |
| AMK48226.1 | nonribosomal\_peptide\_synthetase | BGC0001351 | Cahuitamycins\_biosynthetic\_gene\_cluster | 27 | 59.7765363128 | 252 | 3.4e-66 |
| BAO98812.1 | putative\_nonribosomal\_peptide\_synthetase | BGC0001002 | Jawsamycin\_biosynthetic\_gene\_cluster | 36 | 30.2917442582 | 252 | 4.5e-66 |
| AGM14933.1 | xantholysin\_synthetase\_B | BGC0000463 | Xantholysin\_biosynthetic\_gene\_cluster | 30 | 50.6517690875 | 252 | 4.5e-66 |
| AHH53508.1 | non-ribosomal\_peptide\_synthetase | BGC0000439 | Taromycin\_biosynthetic\_gene\_cluster | 27 | 62.1973929236 | 252 | 4.5e-66 |
| AGN74885.1 | nonribosomal\_peptide\_synthetase | BGC0000459 | Griseoviridin\_/\_viridogrisein\_biosynthetic\_gene\_cluster | 24 | 82.5574177529 | 251 | 5.9e-66 |
| AEA30274.1 | peptide\_synthetase | BGC0000429 | Skyllamycin\_biosynthetic\_gene\_cluster | 26 | 59.4040968343 | 251 | 7.7e-66 |
| AAF08796.1 | MycB | BGC0001103 | Mycosubtilin\_biosynthetic\_gene\_cluster | 24 | 69.7703289882 | 250 | 1e-65 |
| CCJ67637.1 | TaaB | BGC0000447 | Tolaasin\_biosynthetic\_gene\_cluster | 29 | 50.9000620732 | 250 | 1e-65 |
| ACZ55945.1 | non-ribosomal\_peptide\_synthetase | BGC0000302 | Anabaenopeptin\_biosynthetic\_gene\_cluster | 26 | 63.3767846058 | 250 | 1e-65 |
| ADM34138.1 | non-ribosomal\_peptide\_synthetase | BGC0001084 | Notoamide\_/\_stephacidin\_biosynthetic\_gene\_cluster | 25 | 75.6672873991 | 250 | 1.3e-65 |
| CBD77746.1 | non-ribosomal\_peptide\_synthetase/polyketide\_synthase | BGC0000974 | Crocacin\_biosynthetic\_gene\_cluster | 31 | 40.1613904407 | 250 | 1.3e-65 |
| AAU39361.1 | lichenysin\_synthase\_LchAC | BGC0000381 | Lichenysin\_biosynthetic\_gene\_cluster | 26 | 58.2867783985 | 250 | 1.7e-65 |
| OAQ83772.1 | nonribosomal\_peptide\_synthase | BGC0001358 | Leucinostatins\_biosynthetic\_gene\_cluster | 24 | 64.1837368094 | 249 | 2.2e-65 |
| CAJ46691.1 | non\_ribosomal\_peptide\_synthase | BGC0000969 | Chondramide\_biosynthetic\_gene\_cluster | 31 | 42.3339540658 | 249 | 2.2e-65 |
| AAY91420.2 | non-ribosomal\_peptide\_synthetase\_OfaB | BGC0000399 | Orfamide\_biosynthetic\_gene\_cluster | 29 | 50.713842334 | 249 | 2.9e-65 |
| ABS74179.1 | BmyC | BGC0001090 | Bacillomycin\_biosynthetic\_gene\_cluster | 25 | 52.8243327126 | 249 | 2.9e-65 |
| ANQ43348.1 | non-ribosomal\_peptide\_synthetase | BGC0001347\_c2 | Poaeamide\_B\_biosynthetic\_gene\_cluster | 29 | 50.4655493482 | 248 | 3.8e-65 |
| CAJ77715.1 | Mps1\_protein | BGC0000364 | Glycopeptidolipid\_biosynthetic\_gene\_cluster | 26 | 68.5288640596 | 248 | 3.8e-65 |
| CCJ67636.1 | TaaA | BGC0000447 | Tolaasin\_biosynthetic\_gene\_cluster | 29 | 52.0794537554 | 248 | 5e-65 |
| CAN89656.1 | putative\_non-ribosomal\_peptide\_synthetase | BGC0001070 | Kirromycin\_biosynthetic\_gene\_cluster | 25 | 84.8541278709 | 248 | 5e-65 |
| CBW54672.1 | non\_ribosomal\_peptide\_synthetase | BGC0000971 | Cinnabaramide\_biosynthetic\_gene\_cluster | 32 | 37.9888268156 | 248 | 5e-65 |
| ABD65957.1 | nonribosomal\_peptide\_synthetase | BGC0000341 | Enduracidin\_biosynthetic\_gene\_cluster | 28 | 55.8038485413 | 248 | 6.5e-65 |
| AHB82069.1 | non\_ribosomal\_peptide\_synthetase | BGC0001231 | Microsclerodermins\_biosynthetic\_gene\_cluster | 26 | 59.9627560521 | 248 | 6.5e-65 |
| BAN91483.1 | putative\_Aculeacin\_A\_nonribosomal\_peptide\_synthase | BGC0001220 | Aculeacin\_A\_biosynthetic\_gene\_cluster | 25 | 74.4258224705 | 247 | 8.5e-65 |
| AHD05677.1 | nonribosomal\_peptide\_ligase\_subunit | BGC0000402 | Paenilarvins\_biosynthetic\_gene\_cluster | 24 | 58.9075108628 | 247 | 1.1e-64 |
| ALK27916.1 | non-ribosomal\_peptide\_synthase | BGC0001233 | Feglymycin\_biosynthetic\_gene\_cluster | 25 | 85.5990068281 | 246 | 1.9e-64 |
| AMM63162.1 | AniA | BGC0001371 | Echinocandin\_B\_biosynthetic\_gene\_cluster | 23 | 82.0608317815 | 246 | 1.9e-64 |
| AFJ23825.1 | WLIP\_synthetase\_B | BGC0000462\_c2 | WLIP\_biosynthetic\_gene\_cluster | 28 | 50.4034761018 | 246 | 2.5e-64 |
| EAL89049.1 | nonribosomal\_peptide\_synthase,\_putative | BGC0000355 | Fumiquinazolines\_biosynthetic\_gene\_cluster | 24 | 87.0887647424 | 246 | 2.5e-64 |
| CBD77749.1 | non-ribosomal\_peptide\_synthetase | BGC0000974 | Crocacin\_biosynthetic\_gene\_cluster | 30 | 42.2098075729 | 245 | 4.2e-64 |
| CAQ71829.1 | non\_ribosomal\_peptide\_synthase,\_antibiotic\_synthesis;\_contains\_4\_condensation\_domains,\_3\_AMP-acid\_ligases\_II\_domains,\_3\_PP-binding,\_Phosphopantetheine\_attachment\_site\_and\_a\_putative\_thioesterase\_domain | BGC0001189 | Taiwachelin\_biosynthetic\_gene\_cluster | 24 | 75.9776536313 | 245 | 4.2e-64 |
| AEW31022.1 | plipastatin\_synthetase | BGC0000407 | Plipastatin\_biosynthetic\_gene\_cluster | 26 | 65.3631284916 | 245 | 5.5e-64 |
| AEI70245.1 | nonribosomal\_peptide\_synthetase\_NRPS | BGC0000401 | Paenibactin\_biosynthetic\_gene\_cluster | 27 | 61.8249534451 | 245 | 5.5e-64 |
| ALK27915.1 | non-ribosomal\_peptide\_synthase | BGC0001233 | Feglymycin\_biosynthetic\_gene\_cluster | 24 | 84.8541278709 | 244 | 7.2e-64 |
| CCJ67638.1 | TaaC | BGC0000447 | Tolaasin\_biosynthetic\_gene\_cluster | 31 | 35.8162631906 | 244 | 9.4e-64 |
| AJW76709.1 | DsaG | BGC0001196 | Desotamide\_biosynthetic\_gene\_cluster | 28 | 47.9205462446 | 243 | 1.2e-63 |
| AAR87760.2 | ZmaK | BGC0001059 | Zwittermycin\_A\_biosynthetic\_gene\_cluster | 25 | 51.148355059 | 243 | 1.2e-63 |
| ACM68690.1 | AerG1 | BGC0000298 | Aeruginosin\_biosynthetic\_gene\_cluster | 25 | 58.4729981378 | 243 | 1.6e-63 |
| BAH43869.1 | linear\_pentadecapeptide\_gramicidin\_synthetase\_LgrA | BGC0000367 | Gramicidin\_biosynthetic\_gene\_cluster | 25 | 68.9633767846 | 243 | 1.6e-63 |
| CAE53352.1 | non-ribosomal\_peptide\_synthetase | BGC0000440 | Teicoplanin\_biosynthetic\_gene\_cluster | 27 | 55.2451893234 | 243 | 2.1e-63 |
| AJW76711.1 | DsaI | BGC0001196 | Desotamide\_biosynthetic\_gene\_cluster | 29 | 47.548106766 | 243 | 2.1e-63 |
| CAG15011.1 | peptide\_synthetase,\_module\_4-6 | BGC0000441 | Teicoplanin\_biosynthetic\_gene\_cluster | 27 | 55.2451893234 | 242 | 2.7e-63 |
| AHB82056.1 | non\_ribosomal\_peptide\_synthetase | BGC0001019 | Microsclerodermins\_biosynthetic\_gene\_cluster | 25 | 60.3351955307 | 242 | 2.7e-63 |
| ABL74936.1 | TlmX | BGC0001048 | Tallysomycin\_biosynthetic\_gene\_cluster | 31 | 38.0509000621 | 242 | 2.7e-63 |
| CAE53350.1 | non-ribosomal\_peptide\_synthetase | BGC0000440 | Teicoplanin\_biosynthetic\_gene\_cluster | 35 | 32.7746741155 | 242 | 3.6e-63 |
| AHZ20773.1 | non-ribosomal\_peptide\_synthase | BGC0000369 | Hassallidins\_biosynthetic\_gene\_cluster | 25 | 72.4394785847 | 242 | 4.6e-63 |
| CAG15009.1 | peptide\_synthetase,\_module\_1-2 | BGC0000441 | Teicoplanin\_biosynthetic\_gene\_cluster | 35 | 32.7746741155 | 241 | 6.1e-63 |
| ABX37382.1 | amino\_acid\_adenylation\_domain\_protein | BGC0000984 | Delftibactin\_biosynthetic\_gene\_cluster | 24 | 79.2054624457 | 241 | 6.1e-63 |
| CAE02633.1 | surfactin\_synthetase\_C | BGC0000433 | Surfactin\_biosynthetic\_gene\_cluster | 25 | 58.0384854128 | 241 | 6.1e-63 |
| AAY91419.3 | non-ribosomal\_peptide\_synthetase\_OfaA | BGC0000399 | Orfamide\_biosynthetic\_gene\_cluster | 32 | 36.8094351335 | 241 | 7.9e-63 |
| CAC48361.1 | peptide\_synthetase | BGC0000311 | Balhimycin\_biosynthetic\_gene\_cluster | 26 | 55.183116077 | 241 | 7.9e-63 |
| ABC35522.1 | thiotemplate\_mechanism\_natural\_product\_synthetase | BGC0000186 | Thailandamide\_biosynthetic\_gene\_cluster | 28 | 51.582867784 | 241 | 7.9e-63 |
| ABW17377.1 | PsoC | BGC0000411 | Putisolvin\_biosynthetic\_gene\_cluster | 26 | 58.2867783985 | 240 | 1.4e-62 |
| CBG75492.1 | putative\_NRPS/siderophore\_biosynthesis\_protein | BGC0000423 | Scabichelin\_biosynthetic\_gene\_cluster | 27 | 66.0459342024 | 240 | 1.4e-62 |
| AGC09528.1 | NRPS | BGC0001183 | Lobophorin\_biosynthetic\_gene\_cluster | 27 | 53.2588454376 | 240 | 1.4e-62 |
| ABG94125.1 | non-ribosomal\_peptide\_synthetase | BGC0000417 | Rhodochelin\_biosynthetic\_gene\_cluster | 26 | 68.5288640596 | 240 | 1.4e-62 |
| ACA97577.1 | PmxB | BGC0000408 | Polymyxin\_biosynthetic\_gene\_cluster | 28 | 41.4649286158 | 239 | 2.3e-62 |
| AGA37267.1 | NRPS | BGC0000816 | Malbrancheamide\_biosynthetic\_gene\_cluster | 25 | 68.8392302917 | 239 | 2.3e-62 |
| ABC36785.1 | peptide\_synthetase,\_putative | BGC0000964 | Burkholdac\_biosynthetic\_gene\_cluster | 27 | 59.0316573557 | 239 | 3e-62 |
| BAB69699.1 | iturin\_A\_synthetase\_B | BGC0001098 | Iturin\_biosynthetic\_gene\_cluster | 24 | 61.3283674736 | 238 | 3.9e-62 |
| ctg1\_orf8 |  | BGC0001109 | Pederin\_biosynthetic\_gene\_cluster | 24 | 76.225946617 | 238 | 3.9e-62 |
| AAS47562.1 | mixed\_type\_I\_polyketide\_synthase\_-\_peptide\_synthetase | BGC0001108 | Pederin\_biosynthetic\_gene\_cluster | 24 | 76.225946617 | 238 | 3.9e-62 |
| EFL06865.1 | hypothetical\_protein | BGC0000300 | Amychelin\_biosynthetic\_gene\_cluster | 29 | 51.148355059 | 238 | 5.1e-62 |
| AHD05618.1 | putative\_non-ribosomal\_peptide\_ligase\_domain\_protein | BGC0001033 | Paenilamicin\_biosynthetic\_gene\_cluster | 26 | 59.7765363128 | 238 | 5.1e-62 |
| AHD05621.1 | non-ribosomal\_peptide\_ligase\_domain\_protein | BGC0001033 | Paenilamicin\_biosynthetic\_gene\_cluster | 25 | 55.3693358163 | 238 | 5.1e-62 |
| ALK27914.1 | non-ribosomal\_peptide\_synthase | BGC0001233 | Feglymycin\_biosynthetic\_gene\_cluster | 30 | 39.3544382371 | 238 | 6.7e-62 |
| CAJ34381.1 | NRPS\_protein | BGC0000445 | Thiocoraline\_biosynthetic\_gene\_cluster | 31 | 37.3060211049 | 238 | 6.7e-62 |
| CAC48369.1 | peptide\_synthetase | BGC0000311 | Balhimycin\_biosynthetic\_gene\_cluster | 31 | 36.0645561763 | 238 | 6.7e-62 |
| EFL06866.1 | predicted\_protein | BGC0000300 | Amychelin\_biosynthetic\_gene\_cluster | 25 | 70.4531346989 | 237 | 8.8e-62 |
| ABS74180.1 | BmyB | BGC0001090 | Bacillomycin\_biosynthetic\_gene\_cluster | 24 | 61.39044072 | 237 | 8.8e-62 |
| sln8 | nonribosomal\_peptide\_synthetase | BGC0001230 | Salinamides\_biosynthetic\_gene\_cluster | 27 | 61.2042209808 | 237 | 1.1e-61 |
| ACM79806.1 | ZmaB | BGC0001059 | Zwittermycin\_A\_biosynthetic\_gene\_cluster | 23 | 75.2948479205 | 237 | 1.1e-61 |
| AFJ23819.1 | WLIP\_synthetase\_A | BGC0000462 | WLIP\_biosynthetic\_gene\_cluster | 31 | 36.3749224084 | 237 | 1.5e-61 |
| CAN89663.1 | putative\_non-ribosomal\_peptide\_synthetase | BGC0001070 | Kirromycin\_biosynthetic\_gene\_cluster | 26 | 59.0316573557 | 237 | 1.5e-61 |
| AAY93445.1 | non-ribosomal\_peptide\_synthetase\_PvdL | BGC0000413 | Pyoverdine\_biosynthetic\_gene\_cluster | 26 | 65.0527622595 | 237 | 1.5e-61 |
| AAN65224.1 | peptide\_synthetase-like\_protein | BGC0000832 | Clorobiocin\_biosynthetic\_gene\_cluster | 30 | 37.7405338299 | 237 | 1.5e-61 |
| ABS74209.1 | FenA | BGC0001095 | Fengycin\_biosynthetic\_gene\_cluster | 29 | 47.9205462446 | 236 | 2e-61 |
| KPN90369.1 | NunE | BGC0001416 | Nunapeptin\_/\_nunamycin\_biosynthetic\_gene\_cluster | 30 | 42.7063935444 | 235 | 4.3e-61 |
| CAJ77695.1 | MPS1\_protein | BGC0000363 | Glycopeptidolipid\_biosynthetic\_gene\_cluster | 31 | 37.1198013656 | 235 | 4.3e-61 |
| ACG60782.1 | NRPS(C/A/PCP/C/A/PCP) | BGC0001058 | Zorbamycin\_biosynthetic\_gene\_cluster | 26 | 57.7281191806 | 235 | 5.7e-61 |
| CAE14173.1 |  | BGC0000383 | Luminmycin\_biosynthetic\_gene\_cluster | 25 | 58.8454376164 | 235 | 5.7e-61 |
| BAH43764.1 | tyrocidine\_synthetase\_I | BGC0000452 | Tyrocidine\_biosynthetic\_gene\_cluster | 25 | 54.3140906269 | 234 | 7.4e-61 |
| AFH75328.1 | nonribosomal\_peptide\_synthetase | BGC0000398 | Orfamide\_biosynthetic\_gene\_cluster | 32 | 38.1750465549 | 234 | 9.7e-61 |
| CAA11773.1 | PCZA361.18 | BGC0000322\_c2 | Chloroeremomycin\_biosynthetic\_gene\_cluster | 31 | 36.5611421477 | 233 | 1.3e-60 |
| ADA69239.2 | trans-AT\_hybrid\_polyketide\_synthase-NRPS | BGC0001071 | Nosperin\_biosynthetic\_gene\_cluster | 26 | 53.320918684 | 233 | 1.7e-60 |
| CAA11796.1 | PCZA363.5 | BGC0000322 | Chloroeremomycin\_biosynthetic\_gene\_cluster | 26 | 61.7628801986 | 233 | 1.7e-60 |
| AJM89734.1 | PmxB | BGC0001192 | Colistin\_biosynthetic\_gene\_cluster | 28 | 40.5338299193 | 233 | 2.2e-60 |
| AAF67501.2 | NovH | BGC0000834 | Novobiocin\_biosynthetic\_gene\_cluster | 30 | 37.4922408442 | 233 | 2.2e-60 |
| AHD05616.1 | putative\_non-ribosomal\_peptide\_ligase\_domain\_protein | BGC0001033 | Paenilamicin\_biosynthetic\_gene\_cluster | 25 | 51.5207945376 | 233 | 2.2e-60 |
| ABM21571.1 | CrpC | BGC0000975 | Cryptophycin\_biosynthetic\_gene\_cluster | 30 | 31.0986964618 | 232 | 3.7e-60 |
| AEZ51517.1 | PmxB | BGC0001153 | Polymyxin\_biosynthetic\_gene\_cluster | 28 | 41.5270018622 | 232 | 4.8e-60 |
| AAT01806.1 | non-ribosomal\_peptide\_synthetase | BGC0000365 | Glycopeptidolipid\_biosynthetic\_gene\_cluster | 26 | 57.9764121664 | 232 | 4.8e-60 |
| BAE98155.1 | putative\_non-ribosomal\_peptide\_synthetase | BGC0000339 | Echinomycin\_biosynthetic\_gene\_cluster | 29 | 48.6033519553 | 231 | 6.3e-60 |
| AAT09804.1 | NocA | BGC0000395 | Nocardicin\_A\_biosynthetic\_gene\_cluster | 31 | 37.3680943513 | 231 | 6.3e-60 |
| AJI44167.1 | long-chain-fatty-acid-CoA\_ligase | BGC0001193 | Thiolutin\_biosynthetic\_gene\_cluster | 26 | 53.6312849162 | 231 | 8.2e-60 |
| AIW82277.1 | PuwA | BGC0001125 | Puwainaphycins\_biosynthetic\_gene\_cluster | 26 | 60.7076350093 | 231 | 8.2e-60 |
| EPE34341.1 | Acetyl-CoA\_synthetase-like\_protein | BGC0001035 | Pneumocandin\_biosynthetic\_gene\_cluster | 23 | 73.6809435133 | 230 | 1.1e-59 |
| AAZ55898.1 | amino\_acid\_adenylation | BGC0000359 | Fuscachelin\_biosynthetic\_gene\_cluster | 32 | 37.8646803228 | 230 | 1.4e-59 |
| CAJ21198.2 | non-ribosomal\_peptide\_synthetase | BGC0000297 | Aeruginoside\_biosynthetic\_gene\_cluster | 26 | 52.7001862197 | 230 | 1.4e-59 |
| AFO85453.1 | non-ribosomal\_peptide\_synthetase | BGC0000391 | Methylpendolmycin/pendolmycin\_biosynthetic\_gene\_cluster | 29 | 39.47858473 | 230 | 1.8e-59 |
| CCM44336.1 | putative\_nonribosomal\_peptide\_synthetase | BGC0001056 | Zeamine\_biosynthetic\_gene\_cluster | 24 | 80.3227808814 | 230 | 1.8e-59 |
| CBA63680.1 | nonribosomal\_peptide\_synthetase\_NRPS | BGC0000368 | Griseobactin\_biosynthetic\_gene\_cluster | 31 | 39.1061452514 | 230 | 1.8e-59 |
| AGD80618.1 | non-ribosomal\_peptide\_synthetase | BGC0000394 | Naphthyridinomycin\_biosynthetic\_gene\_cluster | 31 | 36.8094351335 | 229 | 2.4e-59 |
| CAM02313.1 | putative\_non-ribosomal\_peptide\_synthetase | BGC0000349 | Erythrochelin\_biosynthetic\_gene\_cluster | 29 | 47.7343265053 | 229 | 2.4e-59 |
| AKP45399.1 | CysK | BGC0001413 | Cystobactamide\_biosynthetic\_gene\_cluster | 25 | 81.6883923029 | 229 | 3.1e-59 |
| CAK15815.1 | putative\_non\_ribosomal\_peptide\_synthetase | BGC0000344 | Entolysin\_biosynthetic\_gene\_cluster | 31 | 36.2507759156 | 229 | 3.1e-59 |
| EFL06867.1 | predicted\_protein | BGC0000300 | Amychelin\_biosynthetic\_gene\_cluster | 25 | 62.5698324022 | 229 | 3.1e-59 |
| ALG65319.1 | Cal17 | BGC0001297 | WS9326\_biosynthetic\_gene\_cluster | 25 | 69.2116697703 | 228 | 4.1e-59 |
| EWS95122.1 | hypothetical\_protein | BGC0000306 | Arylomycin\_biosynthetic\_gene\_cluster | 26 | 60.8938547486 | 228 | 4.1e-59 |
| ABX37383.1 | amino\_acid\_adenylation\_domain\_protein | BGC0000984 | Delftibactin\_biosynthetic\_gene\_cluster | 29 | 42.0235878336 | 228 | 5.3e-59 |
| CAJ34382.1 | NRPS\_protein | BGC0000445 | Thiocoraline\_biosynthetic\_gene\_cluster | 26 | 59.2799503414 | 228 | 5.3e-59 |
| AEG64689.1 | LpmA | BGC0000379 | Laspartomycin\_biosynthetic\_gene\_cluster | 31 | 37.3680943513 | 228 | 5.3e-59 |
| AAT09805.1 | NocB | BGC0000395 | Nocardicin\_A\_biosynthetic\_gene\_cluster | 26 | 58.8454376164 | 228 | 6.9e-59 |
| AEI58879.1 | peptide\_synthetase | BGC0000455 | Vancomycin\_biosynthetic\_gene\_cluster | 30 | 35.6300434513 | 228 | 6.9e-59 |
| AIG79224.1 | Hypothetical\_protein | BGC0000419 | Ristomycin\_A\_biosynthetic\_gene\_cluster | 30 | 35.8162631906 | 227 | 9.1e-59 |
| AKC91856.1 | nonribosomal\_peptide\_synthetase | BGC0001414 | Griselimycin\_biosynthetic\_gene\_cluster | 35 | 28.9261328367 | 227 | 9.1e-59 |
| BAC67534.2 | arthrofactin\_synthetase\_A | BGC0000305 | Arthrofactin\_biosynthetic\_gene\_cluster | 29 | 41.4649286158 | 227 | 9.1e-59 |
| CAE52334.1 | non-ribosomal\_peptide\_synthase | BGC0001088 | Albicidin\_biosynthetic\_gene\_cluster | 26 | 60.7697082557 | 227 | 9.1e-59 |
| EFE73308.1 | non-ribosomal\_peptide\_synthetase | BGC0000431 | Stenothricin\_biosynthetic\_gene\_cluster | 32 | 38.8578522657 | 227 | 9.1e-59 |
| AHZ34239.1 | CipB | BGC0001389 | Cichopeptin\_biosynthetic\_gene\_cluster | 29 | 37.5543140906 | 227 | 1.2e-58 |
| AHD05627.1 | putative\_non-ribosomal\_peptide\_ligase\_domain\_protein | BGC0001033 | Paenilamicin\_biosynthetic\_gene\_cluster | 25 | 59.7144630664 | 227 | 1.5e-58 |
| AAF00962.1 | McyC | BGC0001017 | Microcystin\_biosynthetic\_gene\_cluster | 25 | 57.9764121664 | 227 | 1.5e-58 |
| AAG02364.1 | peptide\_synthetase\_NRPS2-1 | BGC0000963 | Bleomycin\_biosynthetic\_gene\_cluster | 28 | 51.9553072626 | 226 | 2.6e-58 |
| AGZ15459.1 | putative\_non-ribosomal\_peptide\_synthetase | BGC0001036 | Polyoxypeptin\_biosynthetic\_gene\_cluster | 26 | 64.8665425202 | 226 | 2.6e-58 |
| CAN89662.1 | putative\_non-ribosomal\_peptide\_synthetase | BGC0001070 | Kirromycin\_biosynthetic\_gene\_cluster | 32 | 38.4854127871 | 226 | 2.6e-58 |
| CCE88377.1 | non-ribosomal\_peptide\_synthetase/polyketide\_synthase | BGC0001034 | Pellasoren\_biosynthetic\_gene\_cluster | 30 | 36.747361887 | 226 | 2.6e-58 |
| AKP45395.1 | CysG | BGC0001413 | Cystobactamide\_biosynthetic\_gene\_cluster | 24 | 62.5698324022 | 225 | 3.4e-58 |
| ACG60773.1 | NRPS(C/A/PCP) | BGC0001058 | Zorbamycin\_biosynthetic\_gene\_cluster | 27 | 53.0105524519 | 225 | 4.5e-58 |
| ABW17375.1 | PsoA | BGC0000411 | Putisolvin\_biosynthetic\_gene\_cluster | 31 | 38.4854127871 | 224 | 7.7e-58 |
| AAA33023.2 | HC-toxin\_synthetase | BGC0000370 | HC-toxin\_biosynthetic\_gene\_cluster | 26 | 56.7349472377 | 224 | 7.7e-58 |
| ABC34483.1 | nonribosomal\_peptide\_synthetase,\_putative | BGC0000961 | Bactobolin\_biosynthetic\_gene\_cluster | 27 | 58.4109248914 | 224 | 1e-57 |
| AIE77076.1 | peptide\_synthetase | BGC0000418 | Ristocetin\_biosynthetic\_gene\_cluster | 30 | 36.0645561763 | 224 | 1e-57 |
| ABH06369.2 | MassC | BGC0000389 | Massetolide\_A\_biosynthetic\_gene\_cluster | 32 | 35.0713842334 | 224 | 1e-57 |
| BAP05597.1 | CalI | BGC0000967 | Calyculin\_biosynthetic\_gene\_cluster | 29 | 41.8994413408 | 223 | 1.3e-57 |
| AEC14348.1 | nonribosomal\_peptide\_synthetase | BGC0000377 | Koranimine\_biosynthetic\_gene\_cluster | 24 | 51.8932340161 | 223 | 1.7e-57 |
| CCA29203.1 | non-ribosomal\_peptide\_synthetase/polyketide\_synthase | BGC0000955 | Althiomycin\_biosynthetic\_gene\_cluster | 26 | 54.748603352 | 223 | 1.7e-57 |
| AIG79240.1 | Hypothetical\_protein | BGC0000419 | Ristomycin\_A\_biosynthetic\_gene\_cluster | 26 | 61.9490999379 | 223 | 1.7e-57 |
| ABL74945.1 | TlmIV | BGC0001048 | Tallysomycin\_biosynthetic\_gene\_cluster | 28 | 52.2656734947 | 223 | 2.2e-57 |
| AFH75329.1 | nonribosomal\_peptide\_synthetase | BGC0000398 | Orfamide\_biosynthetic\_gene\_cluster | 31 | 38.1129733085 | 222 | 2.9e-57 |
| AAG29780.1 | acyl-CoA\_synthetase | BGC0000833 | Coumermycin\_A1\_biosynthetic\_gene\_cluster | 30 | 37.2439478585 | 222 | 2.9e-57 |
| AIE77060.1 | peptide\_synthetase\_module\_7 | BGC0000418 | Ristocetin\_biosynthetic\_gene\_cluster | 26 | 61.9490999379 | 222 | 3.8e-57 |
| CAC48362.1 | peptide\_synthetase | BGC0000311 | Balhimycin\_biosynthetic\_gene\_cluster | 26 | 61.9490999379 | 222 | 3.8e-57 |
| WP\_010369428.1 | phenylalanine\_racemase | BGC0000314 | Bromoalterochromides\_biosynthetic\_gene\_cluster | 24 | 57.1694599628 | 222 | 5e-57 |
| CAB53322.1 | putative\_peptide\_synthetase | BGC0000325 | Coelichelin\_biosynthetic\_gene\_cluster | 32 | 38.0509000621 | 221 | 6.5e-57 |
| AAM80537.1 | StaC | BGC0000290 | A47934\_biosynthetic\_gene\_cluster | 30 | 38.4233395407 | 221 | 8.5e-57 |
| CCJ67648.1 | JagD | BGC0001127 | Jagaricin\_biosynthetic\_gene\_cluster | 26 | 61.2042209808 | 221 | 8.5e-57 |
| AID65225.1 | nonribosomal\_peptide\_synthetase | BGC0000335 | Cystomanamides\_biosynthetic\_gene\_cluster | 25 | 69.1495965239 | 220 | 1.1e-56 |
| AAN85493.1 | nonribosomal\_peptide\_synthetase | BGC0001101 | Leinamycin\_biosynthetic\_gene\_cluster | 25 | 60.0248292986 | 220 | 1.1e-56 |
| AAY93356.2 | non-ribosomal\_peptide\_synthetase\_PvdI | BGC0000413 | Pyoverdine\_biosynthetic\_gene\_cluster | 26 | 59.217877095 | 220 | 1.1e-56 |
| ABD65956.1 | nonribosomal\_peptide\_synthetase | BGC0000341 | Enduracidin\_biosynthetic\_gene\_cluster | 26 | 59.9627560521 | 220 | 1.4e-56 |
| BAH22762.1 | nonribosomal\_peptide\_synthetase | BGC0001018 | Micropeptin\_biosynthetic\_gene\_cluster | 25 | 52.5760397269 | 220 | 1.4e-56 |
| BAB69700.1 | iturin\_A\_synthetase\_C | BGC0001098 | Iturin\_biosynthetic\_gene\_cluster | 28 | 42.3339540658 | 220 | 1.4e-56 |
| AEC14346.1 | nonribosomal\_peptide\_synthetase | BGC0000377 | Koranimine\_biosynthetic\_gene\_cluster | 23 | 63.687150838 | 220 | 1.4e-56 |
| ABI26078.1 | OciB | BGC0000331 | Cyanopeptin\_biosynthetic\_gene\_cluster | 30 | 31.843575419 | 219 | 2.5e-56 |
| BAD55611.1 | putative\_non-ribosomal\_peptide\_synthetase | BGC0001027 | Nocobactin\_NA\_biosynthetic\_gene\_cluster | 28 | 40.782122905 | 219 | 2.5e-56 |
| CAE53353.1 | non-ribosomal\_peptide\_synthetase | BGC0000440 | Teicoplanin\_biosynthetic\_gene\_cluster | 25 | 66.7908131595 | 219 | 2.5e-56 |
| AAG02356.1 | peptide\_synthetase\_NRPS7 | BGC0000963 | Bleomycin\_biosynthetic\_gene\_cluster | 32 | 38.2991930478 | 219 | 3.2e-56 |
| CBW45648.1 | PI\_synthetase\_2 | BGC0000952 | Pristinamycin\_biosynthetic\_gene\_cluster | 31 | 41.9615145872 | 218 | 4.2e-56 |
| CAJ76290.1 | putative\_non-ribosomal\_peptide\_synthase | BGC0000972 | Colibactin\_biosynthetic\_gene\_cluster | 28 | 42.2718808194 | 218 | 4.2e-56 |
| ABD65960.1 | nonribosomal\_peptide\_synthetase | BGC0000341 | Enduracidin\_biosynthetic\_gene\_cluster | 31 | 40.2234636872 | 218 | 5.5e-56 |
| ABS75232.1 | DhbF | BGC0001185 | Bacillibactin\_biosynthetic\_gene\_cluster | 30 | 38.3612662942 | 218 | 5.5e-56 |
| CAD91212.1 | putative\_non-ribosomal\_peptide\_synthetase,\_modules\_4-6 | BGC0000289 | A40926\_biosynthetic\_gene\_cluster | 30 | 35.6300434513 | 218 | 5.5e-56 |
| CAG15012.1 | peptide\_synthetase,\_module\_7 | BGC0000441 | Teicoplanin\_biosynthetic\_gene\_cluster | 24 | 66.7908131595 | 218 | 5.5e-56 |
| AEI58867.1 | peptide\_synthetase | BGC0000455 | Vancomycin\_biosynthetic\_gene\_cluster | 25 | 66.5425201738 | 218 | 7.2e-56 |
| CAJ88610.1 | putative\_non-ribosomal\_peptide\_synthetase | BGC0000327 | Congocidine\_biosynthetic\_gene\_cluster | 28 | 61.4525139665 | 218 | 7.2e-56 |
| ALG65313.1 | Cal23 | BGC0001297 | WS9326\_biosynthetic\_gene\_cluster | 31 | 37.8646803228 | 218 | 7.2e-56 |
| AAY89051.1 | hybrid\_nonribosomal\_peptide\_synthetase/polyketide\_synthase | BGC0001069 | Chivosazole\_biosynthetic\_gene\_cluster | 28 | 38.0509000621 | 218 | 7.2e-56 |
| AHD05617.1 | putative\_non-ribosomal\_peptide\_ligase\_domain\_protein | BGC0001033 | Paenilamicin\_biosynthetic\_gene\_cluster | 24 | 53.6312849162 | 217 | 9.4e-56 |
| CCJ67647.1 | JagC | BGC0001127 | Jagaricin\_biosynthetic\_gene\_cluster | 29 | 38.3612662942 | 217 | 9.4e-56 |
| CAD32904.2 | non-ribosomal\_peptide\_synthetase\_A | BGC0000354 | Friulimicin\_biosynthetic\_gene\_cluster | 29 | 38.9199255121 | 217 | 1.2e-55 |
| AEI58866.1 | peptide\_synthetase | BGC0000455 | Vancomycin\_biosynthetic\_gene\_cluster | 30 | 37.9267535692 | 216 | 2.7e-55 |
| WP\_051700112.1 | non-ribosomal\_peptide\_synthetase | BGC0001368 | Tambromycin\_biosynthetic\_gene\_cluster | 29 | 42.7063935444 | 216 | 2.7e-55 |
| ABB90279.1 | non-ribosomal\_peptide\_synthetase | BGC0001057 | Zearalenone\_biosynthetic\_gene\_cluster | 25 | 77.0328988206 | 215 | 3.6e-55 |
| ADN26252.1 | peptide\_synthetase | BGC0000951 | Pacidamycin\_biosynthetic\_gene\_cluster | 30 | 38.2991930478 | 215 | 6.1e-55 |
| AAG05693.1 | AmbB | BGC0000287 | 2-amino-4-methoxy-trans-3-butenoic\_acid\_biosynthetic\_gene\_cluster | 30 | 41.6511483551 | 215 | 6.1e-55 |
| CDG12864.1 | non-ribosomal\_peptide\_synthetase | BGC0001415 | Althiomycin\_biosynthetic\_gene\_cluster | 28 | 43.4512725016 | 214 | 7.9e-55 |
| ALV82447.1 | putative\_non-ribosomal\_peptide\_synthetase | BGC0001382 | Svaricin\_biosynthetic\_gene\_cluster | 32 | 38.6716325264 | 214 | 7.9e-55 |
| CCP45167.1 | Peptide\_synthetase\_MbtF\_(peptide\_synthase) | BGC0001021 | Mycobactin\_biosynthetic\_gene\_cluster | 29 | 45.9962756052 | 214 | 7.9e-55 |
| CBJ90287.1 | peptide\_synthetase | BGC0000416 | Rhabdopeptides\_biosynthetic\_gene\_cluster | 32 | 27.6225946617 | 214 | 7.9e-55 |
| CAJ45639.1 | vanchrobactin\_non\_ribosomal\_peptide\_synthetase | BGC0000454 | Vanchrobactin\_biosynthetic\_gene\_cluster | 30 | 37.2439478585 | 213 | 1.4e-54 |
| AKA59447.1 | non-ribosomal\_peptide\_synthetase | BGC0001203 | Clarexpoxcin\_biosynthetic\_gene\_cluster | 26 | 58.4729981378 | 213 | 1.4e-54 |
| ABR67750.1 | CmnG | BGC0000316 | Capreomycin\_biosynthetic\_gene\_cluster | 30 | 37.1198013656 | 213 | 2.3e-54 |
| AHI59109.1 | locillomycin\_synthase\_B | BGC0001005 | Locillomycin\_biosynthetic\_gene\_cluster | 25 | 47.2998137803 | 213 | 2.3e-54 |
| AGZ15460.1 | putative\_non-ribosomal\_peptide\_synthetase | BGC0001036 | Polyoxypeptin\_biosynthetic\_gene\_cluster | 27 | 50.713842334 | 213 | 2.3e-54 |
| BAD55612.1 | non-ribosomal\_peptide\_synthetase | BGC0001027 | Nocobactin\_NA\_biosynthetic\_gene\_cluster | 32 | 37.5543140906 | 213 | 2.3e-54 |
| BAF50720.1 | hybrid\_non\_ribosomal\_peptide\_synthetase-polyketide\_synthase | BGC0001116 | Virginiamycin\_biosynthetic\_gene\_cluster | 32 | 32.278088144 | 212 | 3e-54 |
| ADI59531.1 | CorI | BGC0001091 | Corallopyronin\_biosynthetic\_gene\_cluster | 30 | 37.4301675978 | 212 | 3.9e-54 |
| AGC09519.1 | AMP-dependent\_synthetase/ligase | BGC0001183 | Lobophorin\_biosynthetic\_gene\_cluster | 30 | 38.2371198014 | 212 | 5.1e-54 |
| BAO84868.1 | putative\_non-ribosomal\_peptide\_synthetase | BGC0000414 | Quinocarcin\_biosynthetic\_gene\_cluster | 34 | 31.2228429547 | 211 | 6.7e-54 |
| ABP57749.1 | DepE | BGC0000993 | FK228\_biosynthetic\_gene\_cluster | 26 | 58.7212911235 | 211 | 6.7e-54 |
| ABV56585.1 | KtzE | BGC0000378 | Kutznerides\_biosynthetic\_gene\_cluster | 29 | 42.7684667908 | 211 | 8.8e-54 |
| ABV56604.1 | adenylation\_domain\_protein | BGC0000378 | Kutznerides\_biosynthetic\_gene\_cluster | 30 | 37.7405338299 | 210 | 1.1e-53 |
| EOY45602.1 | non-ribosomal\_peptide\_synthetase | BGC0001168 | Livipeptin\_biosynthetic\_gene\_cluster | 30 | 37.7405338299 | 210 | 1.5e-53 |
| CAQ71828.1 | non\_ribosomal\_peptide\_synthase,\_antibiotic\_synthesis;\_contains\_3\_condensation\_domains,\_2\_AMP-acid\_ligases\_II\_domains,\_2\_PP-binding,\_Phosphopantetheine\_attachment\_site | BGC0001189 | Taiwachelin\_biosynthetic\_gene\_cluster | 28 | 48.417132216 | 210 | 2e-53 |
| CCJ67645.1 | JagA | BGC0001127 | Jagaricin\_biosynthetic\_gene\_cluster | 27 | 55.7417752948 | 209 | 2.6e-53 |
| AAY32966.1 | DszC | BGC0001093 | Disorazole\_biosynthetic\_gene\_cluster | 30 | 38.9199255121 | 208 | 4.4e-53 |
| CAP93139.1 |  | BGC0000357 | Fungisporin\_biosynthetic\_gene\_cluster | 26 | 54.9968963377 | 208 | 5.7e-53 |
| AAP92497.1 | nonribosomal\_peptide\_synthetase | BGC0000458 | Viomycin\_biosynthetic\_gene\_cluster | 31 | 36.747361887 | 208 | 7.4e-53 |
| EFY94493.1 | nonribosomal\_peptide\_synthase\_GliP-like,\_putative | BGC0000337 | Destruxins\_biosynthetic\_gene\_cluster | 25 | 56.2383612663 | 208 | 7.4e-53 |
| AHZ34241.1 | CipD | BGC0001389 | Cichopeptin\_biosynthetic\_gene\_cluster | 29 | 36.5611421477 | 208 | 7.4e-53 |
| AGZ03650.1 | SevA | BGC0000426 | Sevadicin\_biosynthetic\_gene\_cluster | 26 | 37.6784605835 | 207 | 1.3e-52 |
| AGD80616.1 | amino\_acid\_adenylation\_domain-containing\_protein | BGC0000394 | Naphthyridinomycin\_biosynthetic\_gene\_cluster | 33 | 31.0366232154 | 207 | 1.7e-52 |
| ERG36783.1 | phenylalanine\_racemase | BGC0000299 | Alterochromides\_biosynthetic\_gene\_cluster | 26 | 53.0105524519 | 207 | 1.7e-52 |
| AAZ23076.1 | peptide\_synthetase | BGC0000291 | A54145\_biosynthetic\_gene\_cluster | 31 | 36.3749224084 | 206 | 2.2e-52 |
| AAW49318.1 | thaxtomin\_synthetase\_A | BGC0000444 | Thaxtomin\_biosynthetic\_gene\_cluster | 33 | 28.0571073867 | 205 | 4.8e-52 |
| ABS90473.1 | NRPS | BGC0001106 | Oxazolomycin\_biosynthetic\_gene\_cluster | 25 | 59.8386095593 | 205 | 6.3e-52 |
| ABD14712.1 | CesB | BGC0000320 | Cereulide\_biosynthetic\_gene\_cluster | 24 | 58.6592178771 | 205 | 6.3e-52 |
| CAJ18237.2 | non-ribosomal\_peptide\_synthetase\_B | BGC0000354 | Friulimicin\_biosynthetic\_gene\_cluster | 27 | 53.5071384233 | 204 | 1.1e-51 |
| sln6 | nonribosomal\_peptide\_synthetase | BGC0001230 | Salinamides\_biosynthetic\_gene\_cluster | 28 | 38.6716325264 | 204 | 1.1e-51 |
| ABC94347.1 | vicibactin\_biosynthesis\_non-ribosomal\_peptide\_synthase\_protein | BGC0000457 | Vicibactin\_biosynthetic\_gene\_cluster | 24 | 51.8311607697 | 204 | 1.1e-51 |
| EFL02193.1 | amino\_acid\_adenylation\_domain-containing\_protein | BGC0000996 | Frontalamides\_biosynthetic\_gene\_cluster | 26 | 63.8733705773 | 203 | 1.4e-51 |
| EFY94500.1 | non-ribosomal\_peptide\_synthetase | BGC0000337 | Destruxins\_biosynthetic\_gene\_cluster | 25 | 51.582867784 | 203 | 1.4e-51 |
| BAH33409.1 | putative\_non-ribosomal\_peptide\_synthetase | BGC0000371 | Heterobactin\_biosynthetic\_gene\_cluster | 27 | 49.8448168839 | 202 | 3.1e-51 |
| CBJ90359.1 | putative\_Peptide\_synthetase | BGC0000465 | Xenortide\_biosynthetic\_gene\_cluster | 25 | 42.1477343265 | 202 | 4.1e-51 |
| AAC32047.1 | FxbB | BGC0000351 | Exochelin\_biosynthetic\_gene\_cluster | 26 | 49.4723774053 | 202 | 4.1e-51 |
| CCJ67646.1 | JagB | BGC0001127 | Jagaricin\_biosynthetic\_gene\_cluster | 29 | 43.4512725016 | 201 | 5.3e-51 |
| CUI25672.1 | Thiolactomycin\_Hybrid\_PKS-NRPS\_system | BGC0001353 | Thiolactomycin\_biosynthetic\_gene\_cluster | 29 | 38.2371198014 | 200 | 1.6e-50 |
| CAL80821.1 | sylD-like\_NRPS/PKS | BGC0000997 | Glidobactin\_biosynthetic\_gene\_cluster | 28 | 40.782122905 | 200 | 2e-50 |
| BAP16689.1 | nonribosomal\_peptide\_synthetase | BGC0000376 | JBIR-34,\_JBIR-35\_biosynthetic\_gene\_cluster | 28 | 42.0856610801 | 199 | 3.5e-50 |
| CAJ34374.1 | NRPS\_protein | BGC0000445 | Thiocoraline\_biosynthetic\_gene\_cluster | 29 | 45.3755431409 | 199 | 3.5e-50 |
| ABM21572.1 | CrpD | BGC0000975 | Cryptophycin\_biosynthetic\_gene\_cluster | 26 | 48.3550589696 | 198 | 4.5e-50 |
| BAO73252.1 | non-ribosomal\_peptide\_synthetase | BGC0000456 | Verlamelin\_biosynthetic\_gene\_cluster | 27 | 38.5474860335 | 198 | 7.7e-50 |
| BAH22764.1 | nonribosomal\_peptide\_synthetase | BGC0001018 | Micropeptin\_biosynthetic\_gene\_cluster | 28 | 31.7815021726 | 198 | 7.7e-50 |
| CDG12865.1 | non-ribosomal\_peptide\_synthetase | BGC0001415 | Althiomycin\_biosynthetic\_gene\_cluster | 30 | 35.0713842334 | 197 | 1e-49 |
| CBL93718.1 | NRPS\_didomain\_PCP-C | BGC0000360 | GE81112\_biosynthetic\_gene\_cluster | 27 | 58.1005586592 | 197 | 1.3e-49 |
| ADD82940.1 | Bat2 | BGC0001099 | Kalimantacin\_/\_batumin\_biosynthetic\_gene\_cluster | 29 | 39.5406579764 | 196 | 2.2e-49 |
| AID65224.1 | nonribosomal\_peptide\_synthetase | BGC0000335 | Cystomanamides\_biosynthetic\_gene\_cluster | 26 | 52.1415270019 | 196 | 2.9e-49 |
| CAD29794.1 | peptide\_synthetase | BGC0001015 | Microcystin\_biosynthetic\_gene\_cluster | 23 | 88.0198634389 | 195 | 3.8e-49 |
| AAZ23075.1 | peptide\_synthetase | BGC0000291 | A54145\_biosynthetic\_gene\_cluster | 29 | 42.0235878336 | 195 | 3.8e-49 |
| ABG00046.1 | non-ribosomal\_peptide\_synthetase | BGC0000387 | Mangotoxin\_biosynthetic\_gene\_cluster | 29 | 36.3749224084 | 195 | 3.8e-49 |
| ABV56588.1 | KtzH | BGC0000378 | Kutznerides\_biosynthetic\_gene\_cluster | 33 | 31.1607697083 | 195 | 6.5e-49 |
| ABS90476.1 | NRPS | BGC0001106 | Oxazolomycin\_biosynthetic\_gene\_cluster | 28 | 36.9335816263 | 195 | 6.5e-49 |
| ALG65314.1 | Cal22 | BGC0001297 | WS9326\_biosynthetic\_gene\_cluster | 29 | 37.6784605835 | 194 | 8.5e-49 |
| WP\_010369430.1 | Non-ribosomal\_peptide\_synthase | BGC0000314 | Bromoalterochromides\_biosynthetic\_gene\_cluster | 22 | 79.0813159528 | 194 | 8.5e-49 |
| ADG27359.1 | peptide\_synthetase | BGC0000296 | Actinomycin\_biosynthetic\_gene\_cluster | 31 | 33.0229671012 | 194 | 1.1e-48 |
| AAZ03552.1 | McnC | BGC0000332 | Cyanopeptolin\_biosynthetic\_gene\_cluster | 28 | 32.278088144 | 194 | 1.1e-48 |
| EAW16180.1 | nonribosomal\_peptide\_synthase,\_putative | BGC0000293 | Acetylaszonalenin\_biosynthetic\_gene\_cluster | 27 | 43.5754189944 | 193 | 1.9e-48 |
| AAM80536.1 | StaD | BGC0000290 | A47934\_biosynthetic\_gene\_cluster | 28 | 42.5822470515 | 192 | 3.2e-48 |
| BAP16693.1 | nonribosomal\_peptide\_synthetase | BGC0000376 | JBIR-34,\_JBIR-35\_biosynthetic\_gene\_cluster | 29 | 37.6784605835 | 192 | 4.2e-48 |
| CCA89326.1 | mixed\_trans-AT\_type\_I\_polyketide\_synthase/nonribosomal\_peptide\_synthetase | BGC0001111 | Rhizopodins\_biosynthetic\_gene\_cluster | 29 | 38.9199255121 | 192 | 4.2e-48 |
| CAF32362.1 | putative\_non-ribosomal\_peptide\_synthetase | BGC0000712 | Paromomycin\_biosynthetic\_gene\_cluster | 30 | 37.7405338299 | 191 | 7.2e-48 |
| ABS90470.1 | NRPS/PKS | BGC0001106 | Oxazolomycin\_biosynthetic\_gene\_cluster | 31 | 30.8504034761 | 191 | 7.2e-48 |
| CAL69889.1 | RhiB\_protein | BGC0001112 | Rhizoxins\_biosynthetic\_gene\_cluster | 29 | 32.8988206083 | 191 | 9.4e-48 |
| ADQ55475.1 | non-ribosomal\_peptide\_synthase\_module | BGC0000350 | ET-743\_biosynthetic\_gene\_cluster | 29 | 37.4301675978 | 190 | 1.2e-47 |
| AHY86403.1 | non\_ribosomal\_peptide\_synthetase | BGC0000329 | Corpeptin\_biosynthetic\_gene\_cluster | 33 | 27.5605214153 | 190 | 1.2e-47 |
| KFH48607.1 | N-like\_protein | BGC0000317 | Cephalosporin\_biosynthetic\_gene\_cluster | 23 | 81.7504655493 | 190 | 2.1e-47 |
| AEE88284.1 | CurF | BGC0000976 | Curacin\_biosynthetic\_gene\_cluster | 24 | 54.0657976412 | 190 | 2.1e-47 |
| AAT70101.1 | CurF | BGC0001165 | Curacin\_biosynthetic\_gene\_cluster | 24 | 54.0657976412 | 190 | 2.1e-47 |
| CAJ96471.1 | Non-ribosomal\_peptide\_synthetase | BGC0000330 | Cupriachelin\_biosynthetic\_gene\_cluster | 31 | 37.8646803228 | 189 | 2.7e-47 |
| ABA59547.1 | Vlm1 | BGC0000453 | Valinomycin\_biosynthetic\_gene\_cluster | 24 | 73.4947237741 | 188 | 4.7e-47 |
| AAL33758.1 | putative\_non-ribosomal\_peptide\_synthetase | BGC0000421 | Safracin\_biosynthetic\_gene\_cluster | 27 | 39.47858473 | 188 | 6.1e-47 |
| AAF19811.1 | MtaC | BGC0001024 | Myxothiazol\_biosynthetic\_gene\_cluster | 28 | 36.3749224084 | 187 | 1e-46 |
| AED90004.1 | non-ribosomal\_peptide\_synthetase\_ThaC1 | BGC0000443 | Thanamycin\_biosynthetic\_gene\_cluster | 28 | 38.8578522657 | 186 | 1.8e-46 |
| AHB38515.1 | EpnG | BGC0000345 | Eponemycin\_biosynthetic\_gene\_cluster | 26 | 51.582867784 | 186 | 1.8e-46 |
| ABP55169.1 | amino\_acid\_adenylation\_domain | BGC0000150 | Sporolide\_biosynthetic\_gene\_cluster | 28 | 36.6232153942 | 186 | 2.3e-46 |
| CBK62746.1 |  | BGC0001115 | Thiomarinol\_biosynthetic\_gene\_cluster | 28 | 35.6300434513 | 185 | 5.2e-46 |
| ABA70582.1 | alpha-aminoadypil-cysteinyl-valine\_synthetase | BGC0000404 | Penicillin\_biosynthetic\_gene\_cluster | 27 | 36.3749224084 | 185 | 6.7e-46 |
| ABR12615.1 | ACV\_synthetase | BGC0000405 | Penicillin\_biosynthetic\_gene\_cluster | 27 | 36.3749224084 | 185 | 6.7e-46 |
| WP\_051700122.1 | non-ribosomal\_peptide\_synthetase | BGC0001368 | Tambromycin\_biosynthetic\_gene\_cluster | 27 | 40.2855369336 | 183 | 2e-45 |
| WP\_051700105.1 | non-ribosomal\_peptide\_synthetase | BGC0001368 | Tambromycin\_biosynthetic\_gene\_cluster | 27 | 43.6995654873 | 183 | 2.6e-45 |
| ALJ49909.1 | TlmI | BGC0001237 | Thiolactomycin\_biosynthetic\_gene\_cluster | 27 | 39.6027312228 | 182 | 4.4e-45 |
| AEC14349.1 | nonribosomal\_peptide\_synthetase | BGC0000377 | Koranimine\_biosynthetic\_gene\_cluster | 26 | 37.4922408442 | 182 | 4.4e-45 |
| CAQ18839.1 | hybrid\_polyketide\_synthase/nonribosomal\_polypetide\_synthetase | BGC0000954 | Ajudazol\_biosynthetic\_gene\_cluster | 30 | 31.5332091868 | 180 | 1.3e-44 |
| CAO91861.1 | putative\_PKS-NRPS\_protein | BGC0000968 | Chaetoglobosin\_biosynthetic\_gene\_cluster | 28 | 39.044072005 | 180 | 1.7e-44 |
| ACZ66258.1 | APS1 | BGC0000304 | Apicidin\_biosynthetic\_gene\_cluster | 27 | 39.6027312228 | 180 | 2.2e-44 |
| ACM79810.1 | ZmaO | BGC0001059 | Zwittermycin\_A\_biosynthetic\_gene\_cluster | 28 | 33.3333333333 | 178 | 6.3e-44 |
| AAK81825.1 | peptide\_synthetase | BGC0000326 | Complestatin\_biosynthetic\_gene\_cluster | 31 | 28.677839851 | 178 | 6.3e-44 |
| CUI25701.1 | amino\_acid\_adenylation\_domain-containing\_protein\_potentially\_involved\_in\_thiotetronic\_acid\_synthesis | BGC0001352 | Thiotetronate\_Tu\_3010\_biosynthetic\_gene\_cluster | 29 | 35.009310987 | 177 | 1.1e-43 |
| CBZ42140.1 | non-ribosomal\_peptide\_synthetase | BGC0001117 | Himastatin\_biosynthetic\_gene\_cluster | 29 | 36.8715083799 | 177 | 1.1e-43 |
| ADL64235.1 | aureusimine\_non-ribosomal\_peptide\_synthetase | BGC0000308 | Aureusimine\_biosynthetic\_gene\_cluster | 23 | 59.0937306021 | 177 | 1.1e-43 |
| AHB38511.1 | EpnJ | BGC0000345 | Eponemycin\_biosynthetic\_gene\_cluster | 29 | 36.8094351335 | 176 | 2.4e-43 |
| ANI24100.1 | nonribosomal\_peptide\_synthetase | BGC0001235 | Antalid\_biosynthetic\_gene\_cluster | 28 | 31.7194289261 | 175 | 4.1e-43 |
| ACJ04424.1 | aureobasidin\_A1\_biosynthesis\_complex | BGC0000307 | Aureobasidin\_A1\_biosynthetic\_gene\_cluster | 26 | 48.2309124767 | 175 | 7e-43 |
| AAN85506.1 | nonribosomal\_peptide\_synthetase | BGC0001101 | Leinamycin\_biosynthetic\_gene\_cluster | 30 | 37.6163873371 | 173 | 1.6e-42 |
| AGC83576.1 | NRPS | BGC0000818 | Notoamide\_biosynthetic\_gene\_cluster | 28 | 38.4233395407 | 173 | 2.7e-42 |
| ALD82525.1 | non-ribosomal\_peptide\_synthase | BGC0001212 | Nannocystin\_biosynthetic\_gene\_cluster | 29 | 33.0850403476 | 172 | 4.5e-42 |
| AAL33756.1 | putative\_non-ribosomal\_peptide\_synthetase | BGC0000421 | Safracin\_biosynthetic\_gene\_cluster | 26 | 52.886405959 | 171 | 5.9e-42 |
| ADY16697.1 | TqaA | BGC0001142 | Tryptoquialanine\_biosynthetic\_gene\_cluster | 31 | 26.8777157045 | 171 | 5.9e-42 |
| ABX71111.1 | Lct28 | BGC0000238 | Lactonamycin\_biosynthetic\_gene\_cluster | 31 | 31.5952824333 | 171 | 1e-41 |
| WP\_028678148.1 | non-ribosomal\_peptide\_synthetase | BGC0001228 | Retimycin\_biosynthetic\_gene\_cluster | 30 | 27.808814401 | 171 | 1e-41 |
| ACN39728.1 | SibE | BGC0000428 | Sibiromycin\_biosynthetic\_gene\_cluster | 28 | 35.5058969584 | 169 | 2.9e-41 |
| AGC65516.1 | TtbA | BGC0001050 | Thalassospiramide\_biosynthetic\_gene\_cluster | 28 | 38.60955928 | 169 | 3.8e-41 |
| ADH04680.1 | hybrid\_polyketide\_synthase/non-ribosomal\_peptide\_synthetase | BGC0001344 | Tubulysin\_biosynthetic\_gene\_cluster | 29 | 35.1955307263 | 168 | 5e-41 |
| AAR87759.1 | ZmaJ | BGC0001059 | Zwittermycin\_A\_biosynthetic\_gene\_cluster | 27 | 31.5332091868 | 168 | 6.5e-41 |
| CAQ71827.1 | non\_ribosomal\_peptide\_synthase,\_antibiotic\_synthesis;\_contains\_1\_condensation\_domain,\_1\_AMP-acid\_ligases\_II\_domain | BGC0001189 | Taiwachelin\_biosynthetic\_gene\_cluster | 27 | 44.2582247052 | 167 | 1.1e-40 |
| CAG29031.1 | nonribosomal\_peptide\_synthetase\_(modules\_1\_and\_2) | BGC0001023 | Myxochromide\_biosynthetic\_gene\_cluster | 30 | 29.8572315332 | 166 | 1.9e-40 |
| ACR33075.1 | proline\_adenylation\_protein | BGC0000017 | Anatoxin\_biosynthetic\_gene\_cluster | 26 | 33.147113594 | 166 | 2.5e-40 |
| AGZ15476.1 | putative\_non\_ribosomal\_peptide\_synthetase | BGC0001036 | Polyoxypeptin\_biosynthetic\_gene\_cluster | 27 | 36.6852886406 | 166 | 3.2e-40 |
| ABC34137.1 | peptide\_synthetase,\_putative | BGC0000961 | Bactobolin\_biosynthetic\_gene\_cluster | 28 | 32.9608938547 | 166 | 3.2e-40 |
| CAF05648.1 | TubC\_protein | BGC0001053 | Tubulysin\_biosynthetic\_gene\_cluster | 29 | 28.1812538796 | 164 | 9.4e-40 |
| ABB69752.1 | PlaP4 | BGC0000654 | Phenalinolactone\_biosynthetic\_gene\_cluster | 28 | 34.6989447548 | 164 | 1.2e-39 |
| AAF19815.1 | MtaG | BGC0001024 | Myxothiazol\_biosynthetic\_gene\_cluster | 33 | 21.3531967722 | 163 | 1.6e-39 |
| ABL74940.1 | TlmVI | BGC0001048 | Tallysomycin\_biosynthetic\_gene\_cluster | 29 | 33.147113594 | 163 | 2.1e-39 |
| BAI63289.1 | putative\_non-ribosomal\_peptide\_synthetase | BGC0000434 | SW-163\_biosynthetic\_gene\_cluster | 32 | 28.3054003724 | 163 | 2.1e-39 |
| CBJ89771.1 | Non-ribosomal\_peptide\_synthase\_involved\_in\_Xenocoumacin\_synthesis | BGC0001054 | Xenocoumacin\_biosynthetic\_gene\_cluster | 27 | 36.2507759156 | 163 | 2.7e-39 |
| AET98905.1 | putative\_non-ribosomal\_peptide\_synthetase | BGC0000415 | Quinomycin\_biosynthetic\_gene\_cluster | 33 | 27.3122284295 | 162 | 3.6e-39 |
| AAW03327.1 | CtaD | BGC0000982 | Cystothiazole\_A\_biosynthetic\_gene\_cluster | 25 | 40.2234636872 | 162 | 3.6e-39 |
| ABW71852.1 | nonribosomal\_peptide\_synthetase | BGC0000303 | Anthramycin\_biosynthetic\_gene\_cluster | 27 | 38.0509000621 | 161 | 8e-39 |
| AGE11891.1 | nonribosomal\_peptide\_synthetase | BGC0000366 | Gobichelin\_biosynthetic\_gene\_cluster | 29 | 37.6163873371 | 161 | 1e-38 |
| ACK77757.1 | putative\_indigoidine\_synthase\_IndC | BGC0000727 | Auricin\_deoxysugar\_moieties\_biosynthetic\_gene\_cluster | 29 | 28.8019863439 | 161 | 1e-38 |
| BAH04161.1 | putative\_non-ribosomal\_peptide\_synthetase | BGC0000450 | Triostin\_A\_biosynthetic\_gene\_cluster | 33 | 27.3122284295 | 160 | 1.8e-38 |
| ADB02873.1 | AzicV | BGC0000202 | Azicemicin\_biosynthetic\_gene\_cluster | 29 | 31.2849162011 | 160 | 2.3e-38 |
| AFV27434.1 | indigoidine\_synthase | BGC0000375 | Indigoidine\_biosynthetic\_gene\_cluster | 29 | 27.8708876474 | 158 | 5.2e-38 |
| CAE52342.1 | non-ribosomal\_peptide\_synthase | BGC0001088 | Albicidin\_biosynthetic\_gene\_cluster | 31 | 26.1328367474 | 156 | 2e-37 |
| CBL93716.1 | NRPS\_A-domain | BGC0000360 | GE81112\_biosynthetic\_gene\_cluster | 30 | 31.7194289261 | 156 | 3.4e-37 |
| ANY57892.1 | PenN | BGC0001372 | Penigequinolone\_biosynthetic\_gene\_cluster | 26 | 41.4649286158 | 155 | 4.4e-37 |
| EAW09117.1 | hybrid\_NRPS/PKS\_enzyme,\_putative | BGC0000983 | Cytochalasin\_biosynthetic\_gene\_cluster | 25 | 37.8026070764 | 155 | 7.5e-37 |
| BAP34707.1 | AMP-dependent\_synthetase\_and\_ligase | BGC0000078 | Incednine\_biosynthetic\_gene\_cluster | 28 | 30.9124767225 | 154 | 9.8e-37 |
| AAG02359.1 | peptide\_synthetase\_NRPS5-4-3 | BGC0000963 | Bleomycin\_biosynthetic\_gene\_cluster | 29 | 37.6163873371 | 154 | 9.8e-37 |
| ADQ55476.1 | non-ribosomal\_peptide\_synthase\_dimodule | BGC0000350 | ET-743\_biosynthetic\_gene\_cluster | 24 | 37.3680943513 | 153 | 2.2e-36 |
| CAD89775.1 | MelD\_protein | BGC0001010 | Melithiazol\_biosynthetic\_gene\_cluster | 25 | 43.4512725016 | 153 | 2.8e-36 |
| ABM91454.1 | lysergyl\_peptide\_synthetase\_LpsB | BGC0000348 | Ergovaline\_biosynthetic\_gene\_cluster | 29 | 26.939788951 | 153 | 2.8e-36 |
| BAU50944.1 | nonribosomal\_peptide\_synthetase | BGC0001379 | BD-12\_biosynthetic\_gene\_cluster | 30 | 26.5052762259 | 152 | 3.7e-36 |
| CAM59606.1 | non-ribosomal\_peptide\_synthetase | BGC0000297 | Aeruginoside\_biosynthetic\_gene\_cluster | 26 | 35.3196772191 | 152 | 4.8e-36 |
| CAF05647.1 | TubB\_protein | BGC0001053 | Tubulysin\_biosynthetic\_gene\_cluster | 29 | 31.0366232154 | 152 | 4.8e-36 |
| AKD43754.1 | HerJ | BGC0001349 | Heronamide\_biosynthetic\_gene\_cluster | 27 | 30.7262569832 | 151 | 6.3e-36 |
| BAL15726.1 | NRPS | BGC0000432 | Streptothricin\_biosynthetic\_gene\_cluster | 30 | 32.0297951583 | 149 | 2.4e-35 |
| CRG85572.1 | nonribosomal\_peptide\_synthase,\_putative | BGC0001402 | Cyclochlorotine\_biosynthetic\_gene\_cluster | 23 | 52.3898199876 | 149 | 2.4e-35 |
| ALA09365.1 | AMP-dependent\_synthetase\_and\_ligase | BGC0001303 | Lobosamide\_biosynthetic\_gene\_cluster | 27 | 30.2917442582 | 149 | 3.1e-35 |
| XP\_001220460.1 | hypothetical\_protein | BGC0001182 | Chaetoglobosins\_biosynthetic\_gene\_cluster | 23 | 62.5077591558 | 149 | 3.1e-35 |
| AAF86395.1 | FkbP | BGC0000994 | FK520\_biosynthetic\_gene\_cluster | 25 | 41.5890751086 | 148 | 5.4e-35 |
| AAC32048.1 | FxbC | BGC0000351 | Exochelin\_biosynthetic\_gene\_cluster | 29 | 33.4574798262 | 148 | 5.4e-35 |
| CAN89664.1 | putative\_amino\_acid\_adenylating\_enzyme | BGC0001070 | Kirromycin\_biosynthetic\_gene\_cluster | 29 | 30.8504034761 | 147 | 1.6e-34 |
| AGI61652.1 | long\_chain\_fatty\_acid\_CoA\_ligase | BGC0000095\_c2 | Micromonolactam\_biosynthetic\_gene\_cluster | 27 | 31.2849162011 | 147 | 1.6e-34 |
| AAC68816.1 | FK506\_peptide\_synthetase | BGC0000353 | FK506\_biosynthetic\_gene\_cluster | 26 | 40.5959031657 | 144 | 1e-33 |
| AEA29643.1 | putative\_nonribosomal\_peptide\_synthetase | BGC0000409 | Porothramycin\_biosynthetic\_gene\_cluster | 25 | 38.9819987585 | 143 | 1.7e-33 |
| AGC65517.1 | TtbB | BGC0001050 | Thalassospiramide\_biosynthetic\_gene\_cluster | 29 | 30.7883302297 | 142 | 3.8e-33 |
| ACO94492.1 | NRPS\_adenylation\_domain\_protein | BGC0000097 | ML-449\_biosynthetic\_gene\_cluster | 29 | 31.0366232154 | 142 | 3.8e-33 |
| ABP55217.1 | AMP-dependent\_synthetase\_and\_ligase | BGC0000142 | Salinilactam\_biosynthetic\_gene\_cluster | 27 | 30.5400372439 | 142 | 5e-33 |
| ADZ45324.1 | non-ribosomal\_peptide\_synthetase | BGC0001020 | Muraymycin\_biosynthetic\_gene\_cluster | 26 | 36.0024829299 | 141 | 6.5e-33 |
| AEH41789.1 | HrmK | BGC0000374 | Hormaomycin\_biosynthetic\_gene\_cluster | 30 | 32.4643078833 | 141 | 6.5e-33 |
| AFK57220.1 | DidI | BGC0000985 | Didemnin\_biosynthetic\_gene\_cluster | 27 | 33.8299193048 | 138 | 9.5e-32 |
| BAD08370.1 | non-ribosomal\_peptide\_synthetase | BGC0000167 | Vicenistatin\_biosynthetic\_gene\_cluster | 29 | 31.7815021726 | 133 | 1.8e-30 |
| AAY42398.1 | HctF | BGC0001000 | Hectochlorin\_biosynthetic\_gene\_cluster | 22 | 53.0726256983 | 132 | 4e-30 |
| ACO94461.1 | putative\_AMP-dependent\_acyl-CoA\_synthetase/ligase | BGC0000029 | BE-14106\_biosynthetic\_gene\_cluster | 27 | 32.9608938547 | 131 | 1.2e-29 |
| AGA37269.1 | NRPS | BGC0000819 | Paraherquamide\_biosynthetic\_gene\_cluster | 26 | 26.8156424581 | 130 | 2e-29 |
| ACO94489.1 | putative\_AMP-dependent\_acyl-CoA\_synthetase/ligase | BGC0000097 | ML-449\_biosynthetic\_gene\_cluster | 27 | 33.0850403476 | 126 | 2.2e-28 |
| ADN43685.1 | DmbS | BGC0001136 | Desmethylbassianin\_biosynthetic\_gene\_cluster | 26 | 34.0782122905 | 125 | 6.3e-28 |
| AKD43757.1 | HerL | BGC0001349 | Heronamide\_biosynthetic\_gene\_cluster | 29 | 30.8504034761 | 123 | 2.4e-27 |
| CDG76957.1 | non-ribosomal\_peptide\_synthetase | BGC0000446 | Tilivalline\_biosynthetic\_gene\_cluster | 25 | 30.7262569832 | 122 | 4.1e-27 |
| CAG69516.1 | putative\_non-ribosomal\_peptide\_synthetase\_with\_condensation,\_AMP-binding\_and\_phosphopantetheine-binding\_domains | BGC0000352 | Fimsbactin\_biosynthetic\_gene\_cluster | 23 | 31.5952824333 | 114 | 1.5e-24 |
| AAU34203.1 | mannopeptimycin\_peptide\_synthetase\_MppB | BGC0000388 | Mannopeptimycin\_biosynthetic\_gene\_cluster | 23 | 32.5263811297 | 110 | 1.6e-23 |
| AAO39104.1 | AdmJ | BGC0000956 | Andrimid\_biosynthetic\_gene\_cluster | 25 | 31.7815021726 | 108 | 1e-22 |
| BAH22763.1 | nonribosomal\_peptide\_synthetase | BGC0001018 | Micropeptin\_biosynthetic\_gene\_cluster | 22 | 21.6014897579 | 97 | 1.1e-19 |
| AAF01762.1 | AM-toxin\_synthetase | BGC0001261 | AM-toxin\_biosynthetic\_gene\_cluster | 24 | 25.6983240223 | 96 | 2.4e-19 |
| AAZ03551.1 | McnB | BGC0000332 | Cyanopeptolin\_biosynthetic\_gene\_cluster | 22 | 21.6014897579 | 96 | 3.2e-19 |
| AAO39105.1 | AdmK | BGC0000956 | Andrimid\_biosynthetic\_gene\_cluster | 26 | 22.4084419615 | 91 | 1.3e-17 |
| AAF00961.1 | McyB | BGC0001017 | Microcystin\_biosynthetic\_gene\_cluster | 24 | 21.9739292365 | 89 | 3.9e-17 |
| ctg1\_orf000000 |  | BGC0000900 | Ferrichrome\_biosynthetic\_gene\_cluster | 24 | 26.4432029795 | 85 | 7.3e-16 |
| AAX98210.1 | acyl\_CoA\_ligase | BGC0000052 | ECO-02301\_biosynthetic\_gene\_cluster | 24 | 31.6573556797 | 74 | 1.3e-12 |
| CCA54214.1 | Long-chain-fatty-acid--CoA\_ligase | BGC0000893 | Chloramphenicol\_biosynthetic\_gene\_cluster | 22 | 31.4711359404 | 60 | 1.9e-08 |
